# Supplementary material for: Ursodeoxycholic acid as a novel disease-modifying treatment for Parkinson’s disease: protocol for a two-centre, randomised, double-blind, placebo-controlled trial, The 'UP' study
Source: BMJ Open. 2020 Aug 5;10(8):e038911. doi: 10.1136/bmjopen-2020-038911 (PMC7409998; doi:10.1136/bmjopen-2020-038911)
Supplement: Author's manuscript [file bmjopen-2020-038911.draft_revisions.pdf]

BMJ Open is committed to open peer review. As part of this commitment we make the peer review history of every article we publish publicly available.

When an article is published we post the peer reviewers' comments and the authors' responses online. We also post the versions of the paper that were used during peer review. These are the versions that the peer review comments apply to.

The versions of the paper that follow are the versions that were submitted during the peer review process. They are not the versions of record or the final published versions. They should not be cited or distributed as the published version of this manuscript.

BMJ Open is an open access journal and the full, final, typeset and author-corrected version of record of the manuscript is available on our site with no access controls, subscription charges or pay-per-view fees (http://bmjopen.bmj.com).

If you have any questions on BMJ Open's open peer review process please email info.bmjopen@bmj.com

BMJ Open

The UP Study – Ursodeoxycholic acid as a novel disease-modifying treatment for Parkinson's disease: Protocol for a two-centre, randomized, double-blind, placebo-controlled trial

Journal:	*BMJ Open*
Manuscript ID	bmjopen-2020-038911
Article Type:	Protocol
Date Submitted by the Author:	28-Mar-2020

Complete List of Authors:	Payne, Thomas; The University of Sheffield Institute for Translational Neuroscience; NIHR Sheffield Biomedical Research Centre<br>Sassani, Matilde; The University of Sheffield Institute for Translational Neuroscience<br>Buckley, Ellen; NIHR Sheffield Biomedical Research Centre; The University of Sheffield, Institute for In Silico Medicine<br>Moll, Sarah; NIHR Sheffield Biomedical Research Centre<br>Anton, Adriana; NIHR Sheffield Biomedical Research Centre; The University of Sheffield, Academic Unit of Radiology<br>Appleby, Matthew; University College London Institute of Neurology<br>Maru, Seema; University College London Institute of Neurology<br>McNeill, Alisdair; The University of Sheffield Institute for Translational Neuroscience<br>Hoggard, N; The University of Sheffield, Academic Unit of Radiology<br>Mazza, Claudia; The University of Sheffield, Institute for In Silico Medicine<br>Wilkinson, Iain; The University of Sheffield, Academic Unit of Radiology<br>Jenkins, Thomas; The University of Sheffield Institute for Translational Neuroscience<br>Foltynie, Thomas; University College London Institute of Neurology<br>Bandmann, O; The University of Sheffield Institute for Translational Neuroscience; NIHR Sheffield Biomedical Research Centre

Keywords:	Parkinson-s disease < NEUROLOGY, Neurology < INTERNAL MEDICINE, Magnetic resonance imaging < RADIOLOGY & IMAGING

SCHOLARONE™
Manuscripts

I, the Submitting Author has the right to grant and does grant on behalf of all authors of the Work (as defined in the below author licence), an exclusive licence and/or a non-exclusive licence for contributions from authors who are: i) UK Crown employees; ii) where BMJ has agreed a CC-BY licence shall apply, and/or iii) in accordance with the terms applicable for US Federal Government officers or employees acting as part of their official duties; on a worldwide, perpetual, irrevocable, royalty-free basis to BMJ Publishing Group Ltd ("BMJ") its licensees and where the relevant Journal is co-owned by BMJ to the co-owners of the Journal, to publish the Work in this journal and any other BMJ products and to exploit all rights, as set out in our licence.

The Submitting Author accepts and understands that any supply made under these terms is made by BMJ to the Submitting Author unless you are acting as an employee on behalf of your employer or a postgraduate student of an affiliated institution which is paying any applicable article publishing charge ("APC") for Open Access articles. Where the Submitting Author wishes to make the Work available on an Open Access basis (and intends to pay the relevant APC), the terms of reuse of such Open Access shall be governed by a Creative Commons licence – details of these licences and which Creative Commons licence will apply to this Work are set out in our licence referred to above.

Other than as permitted in any relevant BMJ Author's Self Archiving Policies, I confirm this Work has not been accepted for publication elsewhere, is not being considered for publication elsewhere and does not duplicate material already published. I confirm all authors consent to publication of this Work and authorise the granting of this licence.

For peer review only

The UP Study –

Ursodeoxycholic acid as a novel disease-modifying treatment for Parkinson's disease: Protocol for a two-centre, randomized, double-blind, placebo-controlled trial

For peer review only

T. Payne[1,2], M. Sassani[1], E. Buckley[2,3], S. Moll[2], A. Anton[2,4], M. Appleby[5], S. Maru[5], A.McNeill[1,2,3], N. Hoggard[4], C. Mazzà[2,3]; I.D. Willkinson[2,4], T. Jenkins[1,2], T. Foltynie[5], O. Bandmann[1,2]

[1]Sheffield Institute for Translational Neuroscience (SITraN), Dept Neuroscience, University of Sheffield, Sheffield, UK; [2]National Institute for Health Research, Biomedical Research Centre, Royal Hallamshire Hospital, Sheffield, UK, [3]Insigneo Institute for In Silico Medicine, University of Sheffield, Sheffield, UK; [4]Academic Unit of Radiology, University of Sheffield, Sheffield, UK; [5]Institute of Neurology, UCL, London, UK.

Corresponding Author: Prof Oliver Bandmann, Sheffield Institute for Translational Neuroscience, Unviersity of Sheffield, 385a Glossop Road, Sheffield, S10 2HQ.

o.bandmann@sheffield.ac.uk

Word count: 3,905

Version: 1.0 27/3/2020

Abstract

Introduction: There is still no disease modifying treatment for Parkinson's Disease (PD). We had previously undertaken the first drug screen in PD patient tissue and identified Ursodeoxycholic acid (UDCA) as a promising mitochondrial rescue agent. The aims of this trial are to now determine safety and tolerability of UDCA in PD at 30mg/kg, confirm its target engagement in PD patient brain tissue, apply a novel motion-sensor based approach to quantify disease progression objectively, and estimate the mean effect size and its variance on the change in motor severity.

Methods and Analysis: This is a phase II, two-centre, double-blind, randomised, placebo-controlled trial of UDCA at a dose of 30mg/kg in 30 participants with early PD. Treatment duration is 48 weeks, followed by an 8 week washout phase. Randomisation is 2:1 (drug to placebo). Assessments are performed at baseline, week 12, 24, 36, 48 and 56. The primary outcome is safety and tolerability. Secondary outcomes will compare the change between baseline and week 48 using the following three complementing approaches: Clinical assessment, applying the Movement Disorders Society Unified Parkinson's Disease Rating Scale Part III in the practically defined 'OFF' medication state; ^{31}Phosphorus Magnetic Resonance Spectroscopy to assess levels of ATP and relevant metabolites in the brain; and objective quantification of motor impairment, using a validated, motion-sensor based approach. The primary outcome will be reported using descriptive statistics and comparisons between treatment groups. For each secondary outcome the change from baseline will be summarised within treatment groups using summary statistics and appropriate statistical tests assessing for significant differences. All outcomes will use an intention-to-treat analysis population.

Ethics and Dissemination: This trial has been approved by the East of England – Cambridgeshire and Hertfordshire Research Ethics committee. Results will be disseminated in peer-reviewed journals, presentations at scientific meetings and to patients in lay-summary format.

Trial registration: ClinicalTrials.gov: NCT03840005

Strengths and limitations of this study

- This is the first double-blind, randomised, placebo-controlled trial of Ursodeoxycholic Acid (UDCA) in Parkinson's Disease (PD).
- This study uses novel secondary outcomes not previously used in a clinical trial studying PD; namely ^{31}Phosphorus Magnetic Resonance Spectroscopy (^{31}P-MRS) of disease specific regions and detailed, complementary home and clinic-based motor activity and gait analysis.
- ^{31}P-MRS will allow the assessment of mitochondrial dysfunction directly in the substantia nigra, the most severely affected brain area in PD.
- A limitation of the study is the considerable number of capsules patients will have to take; patients will on average be taking an additional nine extra capsules of medication each day through the trial, significantly increasing their 'pill burden'.
- A further limitation is the small sample size of n=30 with 20 patients on UDCA and 10 patients on placebo, it will not be possible to draw firm conclusions about the neuroprotective effect of UDCA in PD. However, the sample size should allow for appropriate power and sample size calculations for a subsequent

For peer review only

definitive Phase IIb/III study to firmly establish or refute a disease modifying effect of UDCA in PD.

For peer review only

INTRODUCTION

Parkinson's Disease (PD) is a progressive neurodegenerative disorder comprising gait impairment, bradykinesia, rigidity and tremor[1]. It is the second most common neurodegenerative disorder and predicted to double in global prevalence between 2005 and 2030[2]. Developing disease modifying therapies is a crucial step in reducing the associated morbidity of PD and to delay the development of late stage complications such as dementia, postural instability and psychosis.

Mitochondrial dysfunction is a key pathogenic mechanism in both sporadic and familial PD and therefore a promising target for disease-modifying therapy[3]. Our group undertook the first drug screen in genetically stratified PD patient tissue[4 5]. This approach identified ursodeoxycholic acid (UDCA) as a particularly promising mitochondrial rescue compound[5]. Other groups demonstrated independently the neuroprotective effect of UDCA and its taurine conjugate TUDCA in the 1-methyl-4-phenyl-1,2,3,6-tetrahydropyridine (MPTP) mouse model and the rotenone rat model of PD[6 7]. UDCA has been in clinical use for decades primarily for primary biliary cholangitis (previously primary biliary cirrhosis) with excellent safety and tolerability at the standard dose of 15mg/kg[8]. UDCA has also been well tolerated at a higher dose of 30 mg/kg over two years in clinical trials for patients with primary sclerosing cholangitis[9]. UDCA is a naturally occurring bile acid but normally only forms 1-3% of total endogenous human bile acids. However, in patients on standard therapeutic doses of UDCA (13-15 mg/kg/day), UDCA may form up to 40% of total bile acids. Intestinal absorption after an oral dose is high with a first-pass clearance of about 50-60%. Plasma levels reach maximum concentrations after 60 minutes after ingestion with another peak at 3 hours[10].

For peer review only

A pharmacokinetic study of UDCA in Motor Neuron Disease demonstrated a significant correlation between serum concentration at one hour post dose and CSF concentration two hours post dose, with most of the variability in CSF concentrations (78%) explained by variability in serum concentrations. Mean CSF concentration post-dose at 15mg/kg was 86.69nmol/L, at 30mg/kg was 114.22nmol/L and 50mg/kg was 191.11 nmol/L[11].

The main objectives of this trial (The UP Study) are to demonstrate the safety and tolerability of UDCA in PD at a dose of 30mg/kg and to explore the effects of UDCA on novel outcome measures such as ^{31}P-MRS and objective quantification of motor impairment, using a sensor-based approach. Additionally, we hope to collect an estimate of the effect size and variance of UDCA on the change in motor severity of PD over 1 year compared to placebo using long-established clinical assessment tools.

For peer review only

METHODS AND ANALYSIS

Design

This is a phase II, two-centre, double-blind, randomised, placebo-controlled trial of 30mg/kg in Ursodeoxycholic acid in early PD. Treatment duration with drug or placebo is 48 weeks in total, followed by an 8 week washout phase. 30 participants will be included. Randomisation is 2:1 in favour of drug to placebo.

Participants

Patients with early PD, as defined by a clinical diagnosis made by a Movement Disorders Specialist within 3 years prior to recruitment and who demonstrate a clear subjective response to dopaminergic medication, confirmed by the treating physician, will be recruited from two sites; Sheffield Teaching Hospitals NHS Trust (STH) and University College London Hospitals NHS Foundation Trust (UCLH). Key inclusion and exclusion criteria can be found in Table 1.

Participants are typically recruited through specialist Movement Disorders Clinics at both trial sites. The trial has also been advertised online by the Parkinson's UK website, the Cure Parkinson's Trust, the Sheffield National Institute for Health-Related Research (NIHR)-Biomedical Research Centre website (NIHR-BRC) and the NIHR Clinical Research Network websites. Trial advertisements direct participants to contact the STH study team to be provided with a Patient Information Sheet (PIS) and a reply slip to confirm ongoing interest and to organise a pre-screening telephone call to confirm eligibility and suitability for the study.

Study visits either take place at the Clinical Research Facility (CRF) of the Royal Hallamshire Hospital, Sheffield, for STH participants or at the Leonard Wolfson Experimental Neurology Centre, Queen Square, London for UCLH participants.

For peer review only

Primary Outcome

The primary outcome for the UP study is to compare the safety and tolerability of UDCA at 30 mg/kg in PD compared to placebo as indicated by the following: the number of serious adverse events (SAEs), number of adverse treatment-reactions and the number of patients completing the study. The safety and tolerability of UDCA in this study will be compared descriptively with the reported safety and tolerability of Exenatide in the Exenatide-PD trial which followed a broadly similar trial design[12].

Secondary Outcomes

The effect of UDCA versus placebo will be assessed as a change from baseline to week 48 for the following secondary outcomes:

1. Clinical assessment using the Movement Disorders Society Unified Parkinson's Disease Rating Scale (MDS-UPDRS) Part 3 motor examination in the practically-defined "OFF" medication state.

2. *In-vivo* measures of high and low energy metabolite levels (including ATP, phosphocreatine and inorganic phosphate) derived from multi-voxel brain ^{31}P-MRS at baseline and week 48.

3. Sensor-based, objective quantification of motor impairment using data collected with wearable sensors both in supervised (OptoGait and Opals systems, Sheffield patients only, Dynaport Movemonitor+, all patients) as well as in unsupervised real-life conditions (Dynaport Movemonitor+, all patients).

Screening Visit

Participants likely to be eligible are invited for a screening visit where all inclusion and exclusion criteria are reviewed. Participants are offered the opportunity to discuss the

For peer review only

trial and have all questions answered after which they will be asked to provide written informed consent before proceeding to further assessment. Participants have a full demographic, medical and concomitant medication history taken and reviewed. A physical examination to confirm the diagnosis of PD and exclude PD ‘mimic’ conditions is performed. A Montreal Cognitive Assessment (MoCA) and Montgomery-Asberg Depression Rating Scale (MADRS) is performed to exclude concurrent dementia or severe active depression [13 14]. Safety bloods (full blood count, urea & electrolytes, liver function tests, blood glucose, HbA1C, lipid profile) and an ECG is performed at the screening visit. If the participant remains eligible, they are provided with an activity monitor (McRoberts, Dynaport MoveMonitor+) to wear for 1 week prior to the baseline visit as described later. For those undergoing ^{31}P-MRS, this is arranged within 1 week before or on the day of the baseline visit, as described later. The baseline visit is completed within 8 weeks of screening.

Baseline Visit, Randomisation and blinding

Randomisation to either active compound or placebo is administered using a centralised, web-based system hosted by epiGenesys (a wholly owned subsidiary of the University of Sheffield) on behalf of the University of Sheffield Clinical Trials Research Unit (CTRU).

MDS-UPDRS Part 3 Motor Examination is performed in the ‘OFF’ state[15]. The practically defined ‘OFF’ state in this study requires participants to not have taken medication for 8 hours in the case of any drug containing Levodopa, or at least 36 hours in the case of longer acting agents such as dopamine agonists or enzyme inhibitors.

For peer review only

The supervised gait analysis is performed using a combination of an instrumented photoelectric walkway system (Microgate, OptoGait) and inertial sensors (APDM, Opal) system as described below.

Participants are then invited to take their usual dopaminergic medication and after a minimum of 60 minutes undergo the following procedures to reassess them in the practically defined ON: MDS-UPDRS Parts 1-4 I in the 'ON' state, Non-motor Symptom Questionnaire (NMS-QUEST) and the 39-Item Parkinson's Disease Questionnaire (PDQ-39)[15-17].

Intervention

All study medication is provided as a white powder in a hard clear gelatine capsule. Placebo and study drug are completely matched with no identifiable differences in taste, appearance or smell. All packaging and labelling is identical. Each capsule of the active drug contains 250mg of UDCA.

Treatment with UDCA is started at a dose of 250mg (one capsule) per day with an increase by 250mg every 3 days until the target dose is reached, which is divided into 3 doses[18]. Most patients are expected to reach their target dose within 3-4 weeks and be on 9-10 capsules per day.

All participants, trial management and medical staff will be blinded to treatment. Participants undergo clinical assessments by the same blinded assessor at each site who is not involved with safety, adverse event (AE) monitoring or dose titration to avoid any assessment bias or accidental unblinding.

For peer review only

Assessment procedures

Following randomisation, a total of 5 further visits are completed at week 12, 24, 36, 48 and 56. At week 48, treatment is completed and all medication returned. A final visit at week 56 for final safety monitoring and outcome measurement completes the study. Week 12 and 36 are purely for safety monitoring and medication supply.

The MDS-UPDRS Part 3 is completed in the practically defined 'ON' state at week 24 and in the 'OFF' state at week 48 and 56. The complete MDS-UPDRS (Parts 1-4) is completed in the 'ON' state at baseline, week 48 and 56.

The ^{31}P-MRS is repeated in the 7 days prior to week 48 for UCLH participants and on the day of the week 48 visit for STH participants. The week-long unsupervised at-home physical activity monitoring is repeated in the 7 days prior to week 48.

The MoCA, NMS-QUEST, PDQ-39 and MADRS are repeated at week 48 and 56.

At each visit, safety bloods (full blood count, urea & electrolytes, liver function tests, blood glucose, HbA1C, lipid profile) are obtained. In addition, at each visit a 20ml serum sample is taken for long term storage and future research. At the baseline visit, blood is taken for genetic analysis, this will be performed using the NeuroChip Assay that assesses for approximately 180,000 genetic variants associated with neurological diseases[19].

A full schedule of activities can be seen in Table 2.

Exploratory Outcomes

The exploratory outcomes will consist of the change between week 48 and 56 in the following: MDS-UPDRS part 3 'OFF' scores, complete MDS-UPDRS (parts 1-4) 'ON' scores, total Levodopa equivalent dose, MoCA, MADRS, NMS-QUEST and PDQ-39. The repeat assessments at week 56 (8 weeks after cessation of the study medication) will help to determine whether there is a sustained effect of UDCA on both motor and

For peer review only

non-motor aspects of PD which would be in keeping with the assumption of a neuroprotective effect. Conversely, a rapid deterioration of these clinical parameters after cessation of the study drug would suggest a symptomatic effect of UDCA.

Sample Size

The primary outcome of interest for this study is the safety and tolerability of UDCA which will be assessed by comparing the rate of Serious Adverse Events (SAEs) in the UDCA and placebo groups, alongside review of adverse treatment reactions and study completion. As the study is a pilot, it is not powered to compare the SAE rate between the groups statistically, but any SAEs in either group will be presented descriptively, the placebo group providing a baseline against which to view any SAEs in the UDCA group. Should this study result in no SAEs then it would be of interest to determine how likely it is that a larger study would find an intolerable rate of SAEs. For this purpose, we will consider the rate of SAEs reported in the Exenatide PD trial to be tolerable and acceptable (i.e. 20%)[12]. In this study, should no SAEs be found in the group receiving UDCA (n=20) then the likelihood that the true SAE rate is less than 20% is 0.990778.

The study has not been powered formally for the secondary or exploratory outcome measures, therefore interpretation will concentrate on observed trends and confidence intervals for estimated differences.

Patient and Public Involvement

Patient representatives have been involved in the design of the study protocol and have contributed to the generation of participant facing study documentation. Recruitment to the study will be aided by both local PD groups and publicised by The

For peer review only

Cure Parkinson's Trust, Parkinson's UK and Michael J Fox Foundation. Results will be disseminated to all participants upon completion of the trial.

OUTCOME MEASURES

Safety Monitoring

At each visit, participants are asked to report any adverse events that have occurred since the previous visit. AEs may also be detected by the study team reviewing the patient or through notification by the participant's primary care physician. All AEs are assessed by a study doctor for their severity, likely relationship to study drug and required action by a study doctor not involved in the blinded assessment of the patient. All SAEs will be recorded and reported to the sponsor regardless of relation to trial treatment within 24 hours. Any suspected unexpected serious adverse reactions (SUSARs) will be reported to the sponsor immediately to allow facilitation of unblinding as necessary. All AEs reported will be reviewed by the Trial Management Group (TMG), Trial Steering Group (TSG) and monitored by an Independent Data Monitoring Committee (IDMC).

Unblinding requests from other clinicians responsible for a patient's care will be handled by the Principal Investigator (PI) at each site. The PI at each site may also choose to unblind in response to reported AEs as they are reported.

In the event that side effects such as diarrhoea do not resolve and become persistent or intolerable then the patient can have their dose adjusted to their last tolerated dose for the remainder of the study.

All participants will be asked to return unused medication, this medication will be counted and recorded to assess compliance.

For peer review only

Motor Measures

The MDS-UPDRS, is currently the most utilised and validated clinical tool to quantify the disease state of an individual with PD[15]. The minimal clinically important difference in the MDS-UPDRS Part 3 is reported to be an improvement of 3.25 points for detecting minimal, but clinically pertinent, improvement and a deterioration of 4.63 points for observing minimal, but clinically pertinent, worsening[20]. Over a period of 5 years MDS-UPDRS Part III scores were observed to increase (deteriorate) by 2.4 points per year[21]. However, rate of decline may still depend on disease stage and a range of other issues; contemporaneous placebo control data therefore remains essential to evaluate potential new therapies.

Neuropsychological Measures

The MoCA is a globally used and validated measure of cognitive impairment and has been used a broad range of neurological diseases and study designs[13]. The MADRS has been validated in PD as a screening tool for major depression[14 22].

Non-motor and Quality of Life Measures

NMS-QUEST is a clinical screening tool that covers a wide range of non-motor symptoms[17]. PDQ-39 is a validated and widely used quality of life questionnaire that covers a range of measures such as emotional wellbeing, activities of daily living and mobility in the context of PD[16].The total equivalent levodopa dose is calculated using calculations and equivalencies generated previously in a systematic review and allows quantitative comparisons between patients on different medication regimes[23].

For peer review only

^{31}Phosphorous Magnetic Resonance Spectroscopy

^{31}P-MRS is experienced by the patient in the same manner as a standard clinical MRI scan. As the metabolites of interest are phosphorus based, it provides the opportunity to investigate key metabolites in bioenergetics such as ATP, phosphocreatine (PCr) and inorganic phosphate (Pi) which all have clear spectroscopic resonances (Figure 1). It is, therefore, an ideal approach to assess mitochondrial function *in-vivo*. Ratio measures such as Pi/ATP and PCr/ATP have been shown to reflect the status of different aspects of oxidative phosphorylation pathways[24].

Two-dimensional Chemical Shift Imaging (CSI) with Image-selected *in vivo* Spectroscopy (ISIS) will be used for spectral spatial localisation[25 26], with a dedicated multi-nuclear MRI system (Ingenia 3.0T, Philips Healthcare, Best, NL) and dual-tuned ^{1}H/^{31}P head coil (Rapid Biomedical, Würzburg, Germany). Standard clinical T1 and T2 weighted imaging will allow the alignment of the two ^{31}P axial CSI sequences as shown in Figure 2. The two sequences will be aligned to obtain spectra from both the putamen (voxels for both anterior and posterior putamen bilaterally) and the midbrain (one voxel for each left and right). This is a clear advantage over alternative techniques that typically utilise surface coils as it allows the localisation of spectra to these specific brain regions typically involved in early PD. Imaging both anatomical regions is of importance since a plausible consequence of mitochondrial dysfunction in PD may be that of retrograde axonal degeneration, therefore spectra from the striatum may show clear mitochondrial dysfunction even in early disease independent of findings in the midbrain. Previous cross-sectional work using a similar ^{31}P-MRS protocol has demonstrated reductions in ATP and PCr in PD compared to controls in both the putamen and midbrain[27]. Additionally, a further study demonstrated that Pi/ATP ratios were increased in PD compared to controls[28].

For peer review only

Details of the acquisition sequences are shown in Table 3. Spectra will be processed in the time domain using jMRUI software v5.2 (http://www.jmrui.eu) and the AMARES algorithm is used to determine the relative area under each peak[29-31]. Analysis of the ^{31}P-MRS data will focus on the change between randomisation and week 48 of normalised amplitudes of ATP, PCr and Pi, and ratio values such as PCr/ATP and Pi/ATP that assess bioenergetic dysfunction. All STH patients will undergo ^{31}P-MRS. UCLH patients are also invited to attend the STH site for ^{31}P-MRS.

Gait Analysis and Activity Monitoring

Physical activity and gait capacity are assessed at two time points, namely prior to/during the baseline visit and prior to/during the week 48 visit at the end of the treatment period.

Physical activity is assessed using home-based "real-life" monitoring for seven consecutive days. A lightweight physical activity monitor (PAM) containing a triaxial accelerometer, gyroscope, digital memory card and a battery (McRoberts, Dynaport Movemonitor+,Netherlands) has been selected for continuous monitoring in all participants. Participants will wear the device for seven consecutive days and complete a diary to quantify their physical activity and gait characteristics within their normal weekly routine in a "real-world" setting.

Gait capacity is assessed during the study visits (Figure 3) using a combination of wearable inertial sensors and an instrumented walkway. Participants complete gait analysis tasks during baseline and week 48 at the respective centre's Clinical Research Facilities (STH and UCLH). There are three short gait tasks. First, participants are asked to complete the 3m Timed Up and Go test walk at self-selected speed. It is an assessment of functional mobility that incorporates transitional actions of standing, turning, and sitting[32 33]. Then participants complete two continuous gait

For peer review only

tasks at self-selected preferred, and fast paced walking speeds. Each trial consists of walking back and forth at least six times along the 8m walkway with periods of quiet standing recorded at the start and end of each trial. At both sites, participants wear the Dynaport Movemonitor+ during instrumented gait tasks. At the Sheffield site, an instrumented 8m walkway (OptoGait, Microgate Corporation, Bolzano, Italy) and a set of inertial sensors (Opals, APDM Inc, Portland, OR, USA) has also been implemented. The instrumented walkway uses bar-mounted LEDs in a two dimensional configuration. The infrared signals transmitted are broken by the movement of the research subject's feet during walking, and various spatiotemporal gait parameters such as step time, stride length, step width and stance time are computed. The system has a spatial resolution of 1cm and a temporal resolution of milliseconds. The data from the inertial sensors will be used to monitor truncal sway during walking and provide a set of additional digitally mobility outcomes associated to the quality of gait (e.g. gait smoothness, variability, symmetry, etc.) [34 35]. The sensors are positioned at both ankles, the lower back (L5), upper back (C7) and forehead. Each sensor contains an accelerometer, gyroscope and magnetometer and records synchronised data wirelessly. Data will be analysed with validated state of the art algorithms, implemented in Matlab[34 36 37].

STATISTICAL ANALYSIS

Analyses will include all randomised patients (an intention to treat (ITT) analysis population). The Primary Outcome of safety and tolerability will be reported using descriptive statistics and comparisons between treatment groups. Demographic and clinical assessment data will be summarised.

For peer review only

For each of the secondary outcomes the change from baseline will be summarised within treatment groups using standard summary statistics (number of participants, mean, standard deviation, median, minimum and maximum) with appropriate statistical tests assessing for significant differences depending upon the distribution of the data and any relevant co-variates.

DATA MANAGEMENT

Data are kept in accordance with God Clinical Practice, the Data Protection Act 2018 and General Data Protection Regulations. Data management is provided by the University of Sheffield Clinical Trials Research Unit (CTRU). All data is entered remotely on to a centralised database held within the CTRU (Prospect) by a research study member at the study site. Access to Prospect is controlled by usernames and encrypted passwords.

All participants are assigned a unique participant ID number at screening that will link all of the clinical information held about them on the study database. The participant ID number is also used in all correspondence between CTRU and participating centres.

ETHICS AND DISSEMINATION

This trial has been approved by the East of England – Cambridgeshire and Hertford Shire Research Ethics committee (Protocol ID: 18/EE/0280) in November 2018. The trial has been registered on ClinicalTrials.gov (ID: NCT03840005). The study will be conducted in accordance with the local R&D approval and the Declaration of Helsinki. The results will be published in a peer reviewed journal and presented at regional, national and international scientific meetings as appropriate. A plain English summary

For peer review only

of the study results will be sent to the study participants once data analysis has been completed. Results of the study may also be presented at meetings of PD support groups or to other relevant lay audiences.

For peer review only

DISCUSSION

We propose a novel study design for early, proof of concept PD neuroprotection trials, combining assessment for safety and tolerability with ^{31}P-MRS-based conformatin of target engagement for bioenergetics pathways and motion-sensor based objective quantification of disease progression. Our study protocol will be particularly powerful for any compound aiming to directly improve mitochondrial function in PD. Additionally, our approach of using ^{31}P-MRS also holds promise to confirm biologically relevant target engagement for compounds aiming at genetically defined upstream targets such as antisense oligonucleotides (ASO) for *LRRK2* or antibody therapy for alpha-synuclein. Mitochondrial dysfunction is a well-recognized aspect of both LRRK2- and alpha-synuclein-associated PD[38 39].

A recent open-label study of UDCA over 6 weeks with an escalating dose up to 50mg/kg in 5 patients with mild to moderate PD found reasonable tolerability and also used ^{31}P-MRS to assess target engagement[40]. However, ^{31}P-MRS imaging data was obtained in only 3 participants and the methodology differed in that a surface coil was used to acquire occipital lobe spectra only.

In-depth sensor-based gait analysis has the potential to overcome the current limitations of the MDS-UPDRS-based clinical assessment[15]. Gait analysis provides a method of quantifying gait disability and postural instability and therefore has potential as an objective motor endpoint for future studies. There is clear evidence that greater axial involvement predicts a poorer outcome in PD with regard to both cognitive decline and postural instability[41]. It is therefore likely that the greatest value in sensor-based analysis is in assessing a combination of spatiotemporal and upper body gait characteristics both in the formal clinical setting but also in exploring real-life mobility through at-home monitoring[34 42 43].

For peer review only

UDCA has previously been trialled in another neurodegenerative disorder, motor neuron disease (MND) at doses of 15, 30 and 50 mg/kg in a total of 18 patients. Patients were treated for 4 weeks. The main adverse events were minor gastrointestinal side effects, graded as mild to moderate. Side effect profiles and frequency were broadly similar between groups without a clear dose correlation[11]. This represents grounds to hypothesise that the primary outcome of safety and tolerability of UDCA at 30 mg/kg in PD will be achievable. We expect completion of the study analysis by July 2021.

For peer review only

Author Contributions

OB is responsible for the overall trial design with contributions from TF. SM led the overall administration and preparation of the trial. TF, SMaru and MA deliver the trial at the UCLH site. TP, MS, AA, NH, IDW and TJ are responsible for the implementation and analysis of the ^{31}P-MRS. EB, AM and CM are responsible for the implementation and analysis of the sensor-based movement analysis tools. TP and EB are responsible for preparing the manuscript under the supervision of OB. All authors have reviewed and commented on this paper. The sponsor has reviewed all participant-facing documents as part of the ethics application (contact Sarah Moll, sarah.moll2@nhs.net, 0114 2712563).There are no competing interests declared by any author.

Funding Statement

The trial is funded by a grant from the JP Moulton Charitable Foundation with additional support from the Sheffield NIHR Biomedical Research Centre (NIHR BRC). TP is funded by the NIHR BRC and The Cure Parkinson's Trust. AA is funded by the Sheffield NIHR BRC. The funding sources had no role in the design of this study and will not have any role during its execution, analyses, interpretation of the data, or decision to submit results.

Licence

I, the Submitting Author has the right to grant and does grant on behalf of all authors of the Work (as defined in the below author licence), an exclusive licence and/or a non-exclusive licence for contributions from authors who are: i) UK Crown employees; ii) where BMJ has agreed a CC-BY licence shall apply, and/or iii) in accordance with the terms applicable for US Federal Government officers or employees acting as part of their official duties; on a worldwide, perpetual, irrevocable, royalty-free basis to BMJ

For peer review only

Publishing Group Ltd ("BMJ") its licensees and where the relevant Journal is co-owned by BMJ to the co-owners of the Journal, to publish the Work in BMJ Open and any other BMJ products and to exploit all rights, as set out in our licence.

The Submitting Author accepts and understands that any supply made under these terms is made by BMJ to the Submitting Author unless you are acting as an employee on behalf of your employer or a postgraduate student of an affiliated institution which is paying any applicable article publishing charge ("APC") for Open Access articles. Where the Submitting Author wishes to make the Work available on an Open Access basis (and intends to pay the relevant APC), the terms of reuse of such Open Access shall be governed by a Creative Commons licence – details of these licences and which Creative Commons licence will apply to this Work are set out in our licence referred to above.

For peer review only

REFERENCES

1. Kalia LV, Lang AE. Parkinson's disease. *The Lancet* 2015;386(9996):896-912. doi: 10.1016/s0140-6736(14)61393-3

2. Dorsey ER, Constantinescu R, Thompson JP, et al. Projected number of people with Parkinson disease in the most populous nations, 2005 through 2030. *Neurology* 2007;68(5):384-6. doi: 10.1212/01.wnl.0000247740.47667.03 [published Online First: 2006/11/04]

3. Schapira AHV, Olanow CW, Greenamyre JT, et al. Slowing of neurodegeneration in Parkinson's disease and Huntington's disease: future therapeutic perspectives. *The Lancet* 2014;384(9942):545-55. doi: 10.1016/s0140-6736(14)61010-2

4. Mortiboys H, Aasly J, Bandmann O. Ursocholanic acid rescues mitochondrial function in common forms of familial Parkinson's disease. *Brain* 2013;136(Pt 10):3038-50. doi: 10.1093/brain/awt224 [published Online First: 2013/09/04]

5. Mortiboys H, Furmston R, Bronstad G, et al. UDCA exerts beneficial effect on mitochondrial dysfunction in LRRK2(G2019S) carriers and in vivo. *Neurology* 2015;85(10):846-52. doi: 10.1212/WNL.0000000000001905 [published Online First: 2015/08/09]

6. Abdelkader NF, Safar MM, Salem HA. Ursodeoxycholic Acid Ameliorates Apoptotic Cascade in the Rotenone Model of Parkinson's Disease: Modulation of Mitochondrial Perturbations. *Mol Neurobiol* 2016;53(2):810-7. doi: 10.1007/s12035-014-9043-8 [published Online First: 2014/12/17]

7. Castro-Caldas M, Carvalho AN, Rodrigues E, et al. Tauroursodeoxycholic acid prevents MPTP-induced dopaminergic cell death in a mouse model of

For peer review only

Parkinson's disease. *Mol Neurobiol* 2012;46(2):475-86. doi: 10.1007/s12035-012-8295-4 [published Online First: 2012/07/10]

8. Goulis J, Leandro G, Burroughs AK. Randomised controlled trials of ursodeoxycholic-acid therapy for primary biliary cirrhosis: a meta-analysis. *The Lancet* 1999;354(9184):1053-60. doi: 10.1016/s0140-6736(98)11293-x

9. Cullen SN, Rust C, Fleming K, et al. High dose ursodeoxycholic acid for the treatment of primary sclerosing cholangitis is safe and effective. *J Hepatol* 2008;48(5):792-800. doi: 10.1016/j.jhep.2007.12.023 [published Online First: 2008/03/04]

10. Ward A, Brogden RN, Heel RC, et al. Ursodeoxycholic acid: a review of its pharmacological properties and therapeutic efficacy. *Drugs* 1984;27(2):95-131. doi: 10.2165/00003495-198427020-00001 [published Online First: 1984/02/01]

11. Parry GJ, Rodrigues CM, Aranha MM, et al. Safety, tolerability, and cerebrospinal fluid penetration of ursodeoxycholic Acid in patients with amyotrophic lateral sclerosis. *Clinical neuropharmacology* 2010;33(1):17-21. doi: 10.1097/WNF.0b013e3181c47569 [published Online First: 2009/11/26]

12. Athauda D, Maclagan K, Skene SS, et al. Exenatide once weekly versus placebo in Parkinson's disease: a randomised, double-blind, placebo-controlled trial. *The Lancet* 2017;390(10103):1664-75. doi: 10.1016/s0140-6736(17)31585-4

13. Nasreddine ZS, Phillips NA, Bedirian V, et al. The Montreal Cognitive Assessment, MoCA: a brief screening tool for mild cognitive impairment. *Journal of the American Geriatrics Society* 2005;53(4):695-9. doi: 10.1111/j.1532-5415.2005.53221.x [published Online First: 2005/04/09]

For peer review only

14. Montgomery SA, Asberg M. A new depression scale designed to be sensitive to change. *Br J Psychiatry* 1979;134:382-9. doi: 10.1192/bjp.134.4.382 [published Online First: 1979/04/01]

15. Goetz CG, Tilley BC, Shaftman SR, et al. Movement Disorder Society-sponsored revision of the Unified Parkinson's Disease Rating Scale (MDS-UPDRS): scale presentation and clinimetric testing results. *Mov Disord* 2008;23(15):2129-70. doi: 10.1002/mds.22340 [published Online First: 2008/11/26]

16. Jenkinson C, Fitzpatrick R, Peto V, et al. The Parkinson's Disease Questionnaire (PDQ-39): development and validation of a Parkinson's disease summary index score. *Age and ageing* 1997;26(5):353-7. doi: 10.1093/ageing/26.5.353 [published Online First: 1997/11/14]

17. Chaudhuri KR, Martinez-Martin P, Schapira AH, et al. International multicenter pilot study of the first comprehensive self-completed nonmotor symptoms questionnaire for Parkinson's disease: the NMSQuest study. *Mov Disord* 2006;21(7):916-23. doi: 10.1002/mds.20844 [published Online First: 2006/03/21]

18. Abbas G, Lindor KD. Pharmacological treatment of biliary cirrhosis with ursodeoxycholic acid. *Expert opinion on pharmacotherapy* 2010;11(3):387-92. doi: 10.1517/14656560903493460 [published Online First: 2010/01/28]

19. Blauwendraat C, Faghri F, Pihlstrom L, et al. NeuroChip, an updated version of the NeuroX genotyping platform to rapidly screen for variants associated with neurological diseases. *Neurobiol Aging* 2017;57:247 e9-47 e13. doi: 10.1016/j.neurobiolaging.2017.05.009 [published Online First: 2017/06/13]

For peer review only

20. Horvath K, Aschermann Z, Acs P, et al. Minimal clinically important difference on the Motor Examination part of MDS-UPDRS. *Parkinsonism Relat Disord* 2015;21(12):1421-6. doi: 10.1016/j.parkreldis.2015.10.006 [published Online First: 2015/11/19]

21. Holden SK, Finseth T, Sillau SH, et al. Progression of MDS-UPDRS Scores Over Five Years in De Novo Parkinson Disease from the Parkinson's Progression Markers Initiative Cohort. *Mov Disord Clin Pract* 2018;5(1):47-53. doi: 10.1002/mdc3.12553 [published Online First: 2018/04/18]

22. Ketharanathan T, Hanwella R, Weerasundera R, et al. Diagnostic Validity and Factor Analysis of Montgomery-Asberg Depression Rating Scale in Parkinson Disease Population. *Journal of geriatric psychiatry and neurology* 2016;29(3):115-9. doi: 10.1177/0891988715606232 [published Online First: 2015/09/24]

23. Tomlinson CL, Stowe R, Patel S, et al. Systematic review of levodopa dose equivalency reporting in Parkinson's disease. *Mov Disord* 2010;25(15):2649-53. doi: 10.1002/mds.23429 [published Online First: 2010/11/12]

24. Iles RA, Stevens AN, Griffiths JR, et al. Phosphorylation status of liver by 31P-n.m.r. spectroscopy, and its implications for metabolic control. A comparison of 31P-n.m.r. spectroscopy (in vivo and in vitro) with chemical and enzymic determinations of ATP, ADP and Pi. *Biochem J* 1985;229(1):141-51. [published Online First: 1985/07/01]

25. Ordidge RJ, Connelly A, Lohman JAB. Image-selected in Vivo spectroscopy (ISIS). A new technique for spatially selective nmr spectroscopy. *Journal of Magnetic Resonance (1969)* 1986;66(2):283-94. doi: https://doi.org/10.1016/0022-2364(86)90031-4

For peer review only

26. Ordidge RJ, Bowley RM, McHale G. A general approach to selection of multiple cubic volume elements using the ISIS technique. *Magnetic Resonance in Medicine* 1988;8(3):323-31. doi: 10.1002/mrm.1910080309

27. Hattingen E, Magerkurth J, Pilatus U, et al. Phosphorus and proton magnetic resonance spectroscopy demonstrates mitochondrial dysfunction in early and advanced Parkinson's disease. *Brain* 2009;132(Pt 12):3285-97. doi: 10.1093/brain/awp293 [published Online First: 2009/12/03]

28. Hu MTM, Taylor-Robinson SD, Chaudhuri KR, et al. Cortical dysfunction in non-demented Parkinson's disease patients: A combined 31P-MRS and 18FDG-PET study. *Brain* 2000;123(2):340-52. doi: 10.1093/brain/123.2.340

29. Vanhamme L, van den Boogaart A, Van Huffel S. Improved Method for Accurate and Efficient Quantification of MRS Data with Use of Prior Knowledge. *Journal of Magnetic Resonance* 1997;129(1):35-43. doi: 10.1006/jmre.1997.1244

30. Stefan D, Cesare FD, Andrasescu A, et al. Quantitation of magnetic resonance spectroscopy signals: the jMRUI software package. *Measurement Science and Technology* 2009;20(10) doi: 10.1088/0957-0233/20/10/104035

31. Naressi A, Couturier C, Devos JM, et al. Java-based graphical user interface for the MRUI quantitation package. *Magnetic Resonance Materials in Physics, Biology and Medicine* 2001;12(2):141. doi: 10.1007/BF02668096

32. Podsiadlo D, Richardson S. The Timed “Up & Go”: A Test of Basic Functional Mobility for Frail Elderly Persons. *Journal of the American Geriatrics Society* 1991;39(2):142-48. doi: 10.1111/j.1532-5415.1991.tb01616.x

33. van Lummel RC, Walgaard S, Hobert MA, et al. Intra-Rater, Inter-Rater and Test-Retest Reliability of an Instrumented Timed Up and Go (iTUG) Test in

For peer review only

Patients with Parkinson's Disease. *PLoS One* 2016;11(3):e0151881. doi: 10.1371/journal.pone.0151881 [published Online First: 2016/03/22]

34. Buckley C, Galna B, Rochester L, et al. Upper body accelerations as a biomarker of gait impairment in the early stages of Parkinson’s disease. *Gait & Posture* 2019;71:289-95. doi: https://doi.org/10.1016/j.gaitpost.2018.06.166
35. Rehman RZU, Del Din S, Buckley C, et al. Accelerometry-Based Digital Gait Characteristics for Classification of Parkinson's Disease: What Counts? *IEEE Open Journal of Engineering in Medicine and Biology* 2020;1:65-73. doi: 10.1109/ojemb.2020.2966295
36. Del Din S, Godfrey A, Mazza C, et al. Free-living monitoring of Parkinson's disease: Lessons from the field. *Mov Disord* 2016;31(9):1293-313. doi: 10.1002/mds.26718 [published Online First: 2016/07/28]
37. Buckley C, Alcock L, McArdle R, et al. The Role of Movement Analysis in Diagnosing and Monitoring Neurodegenerative Conditions: Insights from Gait and Postural Control. *Brain Sci* 2019;9(2) doi: 10.3390/brainsci9020034 [published Online First: 2019/02/10]
38. Mortiboys H, Johansen KK, Aasly JO, et al. Mitochondrial impairment in patients with Parkinson disease with the G2019S mutation in LRRK2. *Neurology* 2010;75(22):2017-20. doi: 10.1212/WNL.0b013e3181ff9685 [published Online First: 2010/12/01]
39. Di Maio R, Barrett PJ, Hoffman EK, et al. alpha-Synuclein binds to TOM20 and inhibits mitochondrial protein import in Parkinson's disease. *Sci Transl Med* 2016;8(342):342ra78. doi: 10.1126/scitranslmed.aaf3634 [published Online First: 2016/06/10]

40. Sathe AG, Tuite P, Chen C, et al. Pharmacokinetics, Safety, and Tolerability of Orally Administered Ursodeoxycholic Acid in Patients With Parkinson's Disease-A Pilot Study. *J Clin Pharmacol* 2020 doi: 10.1002/jcph.1575 [published Online First: 2020/02/14]

41. Velseboer DC, de Bie RM, Wieske L, et al. Development and external validation of a prognostic model in newly diagnosed Parkinson disease. *Neurology* 2016;86(11):986-93. doi: 10.1212/WNL.0000000000002437 [published Online First: 2016/02/19]

42. Weiss A, Sharifi S, Plotnik M, et al. Toward Automated, At-Home Assessment of Mobility Among Patients With Parkinson Disease, Using a Body-Worn Accelerometer. *Neurorehabilitation and Neural Repair* 2011;25(9):810-18. doi: 10.1177/1545968311424869

43. Morris R, Hickey A, Del Din S, et al. A model of free-living gait: A factor analysis in Parkinson's disease. *Gait & Posture* 2017;52:68-71. doi: https://doi.org/10.1016/j.gaitpost.2016.11.024

44. Hughes AJ, Daniel S, Kilford L, et al. Accuracy of clinical diagnosis of idiopathic Parkinson's disease: a clinico-pathological study of 100 cases. *J Neurol Neurosurg Psychiatry* 1992;55(3):181-84.

For peer review only

Key Inclusion Criteria
• Diagnosis of Parkinson's disease ≤ 3 years ago based on Queen Square Brain Bank criteria [44]<br>• Subjective improvement of motor impairment on dopaminergic medication with confirmation by a movement disorders expert<br>• Hoehn and Yahr stage ≤ 2.5 in the practically defined "ON" medication state<br>• Age 18-75 years of any gender<br>• Able to comply with study protocol and willing to attend necessary study visits<br>• Ability to communicate in English<br>• Ability to take study drug
**Key Exclusion Criteria**
• Diagnosis or suspicion of other cause of parkinsonism<br>• Known abnormality on CT or MRI brain imaging considered likely to compromise compliance with ^{31}Phosphorus MR Spectroscopy acquisition<br>• Known claustrophobia or other reasons why patient could not tolerate or be suitable for MRI<br>• Current or previous exposure to UDCA<br>• Current or previous diagnosis of liver disease, in particular PBC judged to be significant<br>• Prior intracerebral surgical intervention for PD (including deep-brain stimulation)<br>• Already actively participating in a trial of a device, drug or surgical treatment for PD<br>• Participants who lack the capacity to give informed consent<br>• History of alcoholism<br>• Women of child-bearing potential or pregnancy<br>• Concurrent severe depression defined by a score >16 on the Montgomery-Asberg Depression Rating Scale (MADRS)<br>• Concurrent dementia defined by a score lower than 25 on the Montreal Cognitive assessment<br>• Any medical or psychiatric condition which in the investigator's opinion compromises the potential participant's ability to participate<br>• Serum transaminases more than 2 times upper limit of normal<br>• Patients on cyclosporin, nitrendipine or dapsone<br>• Participants with previous or current diagnosis of inflammatory bowel disease

Table 1: Key Inclusion and Exclusion Criteria for the UP Study

For peer review only

Figure 1: Representative ^{31}P-MRS spectra obtained from the midbrain of a healthy volunteer following appropriate phasing and 10Hz Lorentzian apodization. From left to right, phosphomonoesters (PME), inorganic phosphate (Pi), phosphodiesters (PDE), phosphocreatine (PCr), and the three spectral resonances of adenosine triphosphate (γ-,α-,β-ATP).

Figure 2: The substantia nigra slice is placed to cover the midbrain with the highlighted voxels of interest for subsequent analyses highlighted in yellow in the sagittal (A) and axial planes (B). Placement of ^{31}P-MRS slices. The basal ganglia slice is placed over the putamen aligned in both the coronal (C) axial planes (D), and voxels of interest for subsequent analyses are highlighted in yellow. One voxel covers the anterior putamen and another the posterior putamen.

Figure 3: Motion sensor protocols deployed at the two sites. All participants undergo seven day physical activity monitoring in order to estimate physical activity levels and capture temporal and gait quality measures in a real-world setting. In-clinic instrumented gait tasks are also completed at both sites to provide spatiotemporal and gait quality measures of gait capacity. At UCL only red sensor location is implemented.

For peer review only

	Procedure	Screening	Baseline	Week 12	Week 24	Week 36	Week 48	Week 56
Medical History	Consent	X						
	Review inclusion/exclusion criteria	X	X					
	Demographics	X						
	Medical History and Physical Examination	X						
	Height and Weight	X					X	
	Genetics Sample		X					
Medication	Randomisation		X					
	Medication supply		X	X	X	X		
	Concomitant medication review	X	X	X	X	X	X	X
	Compliance review			X	X	X		
Clinical Assessment/Outcome Measures	MDS-UPDRS Part 3 ‘OFF’		X				X	X
	MDS-UPDRS Part 3 ‘ON’				X			
	MDS-UPDRS Parts 1-4 ‘ON’		X				X	X
	MoCA, MADRS	X					X	X
	PDQ-39		X				X	X
	NMS -QUEST		X				X	
Sensor Based Analysi	Dynaport MoveMonitor+ 7 day recording	X					X (7 days prior)	
	OptoGait/Opals gait assessment ‘OFF’		X				X	
MRI	31P-MRS		X				X	
Safety Monitoring	Safety bloods	X	X	X	X	X	X	X
	ECG	X			X			
	AE Review		X	X	X	X	X	X

Table 2: Schedule of activities for The UP Study

For peer review only

Sequence description	Localisation	Decoupling, NOE	TR (ms)	TE (ms)	NSA	Acquired voxel size	Reconstruction matrix	Reconstructed voxel size	Scan duration (min)
^{31}P-Basal Ganglia	^{31}P 2D CSI ISIS localisation	On	4000	0.22	10	40x40x20	12x12	17.5x17.5x20	12:48
^{31}P-Substantia Nigra	^{31}P 2D CSI ISIS localisation	On	4000	0.22	8	40x40x20	14x14	15x15x20	10:16

Table 3: Detailed parameters of the ^{31}P protocol for acquisition. NOE; Nuclear Overhauser Effect, TR; time to repetition, TE; time to echo, NSA; number of signal averages.

For peer review only

World Health Organization Trial Registration Data Set

**First Submitted Date**	February 11, 2019
**First Posted Dat**	February 15, 2019
**Last Update Posted Date**	June 11, 2019
**Actual Study Start Date**	December 18, 2018
**Current Primary Outcome Measures**	• Number of Participants with Incidence of Treatment-Emergent Adverse Events [Time Frame: Timepoint: start of treatment to 56 weeks (visit 6)]<br>Safety of a 56-week UDCA Intervention will be assessed by measuring the number of participants with adverse events that are related to treatment.<br>• Number of Participants with Incidence of Serious Adverse Events [Time Frame: Timepoint: start of treatment to 56 weeks (visit 6)]<br>Safety of a 56-week UDCA Intervention will be assessed by measuring the number of participants with serious adverse events.<br>• Number of Participants that complete the study [Time Frame: Timepoint: start of treatment to 56 weeks (visit 6)]<br>Safety of a 56-week UDCA Intervention will be assessed by measuring the number of participants that complete the study.

For peer review only

**Current Secondary Outcome Measures**	• Mean change from baseline to week 48 in participant scores on the Movement Disorders Society Unified Parkinson's Disease Rating Scale (MDS-UPDRS) part 3 motor subsection in the "OFF" medication state. [Time Frame: Timepoint: 48 weeks (visit 5)]<br>Motor symptoms will be measured using the MDS-UPDRS part 3 motor subsection. Part III of the scale will be completed at baseline, visit 3 (24 weeks), visit 5 (48 weeks). The scale consists of four parts; Part I "Non-motor experiences of daily living" (13 questions), Part II "Motor Experiences of daily living" (13), Part III "Motor Examination" (33) and Part IV "Motor Complications" (6). Each question has five responses that are linked to common clinical terms: 0=Normal, 1=Slight, 2=Mild, 3=Moderate, 4=Severe. Whereas each response is tailored to the question, the progression of impairment is based on consistent infrastructure. "Slight" refers to symptoms with sufficiently low frequency/intensity to cause no impact on function; "Mild" refers to symptoms of frequency/intensity sufficient to cause modest impact on function; "Moderate" refers to symptoms sufficiently frequent/intense to impact considerably, but not prevent, function; "Severe" refers to symptoms that prevent function.<br>• Mean change from baseline to week 48 in in vivo parameter estimates of Adenosine Triphosphate (ATP) levels, derived from participant cranial 31P-Magnetic Resonance Spectroscopy (MRS) centered on the basal ganglia and related motor regions. [Time Frame: Timepoint: 48 weeks (visit 5)]<br>Patients who consent to having the 31P-MR spectroscopy, data will be analysed for the change in energy metabolic levels at baseline and visit 5 (week 48).<br>• Mean change from baseline to week 48 in in vivo parameter estimates of Phosphocreatinine (PCr) levels, derived from participant cranial 31P-Magnetic Resonance Spectroscopy (MRS) centered on the basal ganglia and related motor regions. [Time Frame: Timepoint: 48 weeks (visit 5)]<br>Patients who consent to having the 31P-MR spectroscopy, data will be analysed for the change in energy metabolic levels at baseline and visit 5 (week 48).<br>• Mean change from baseline to week 48 in in vivo parameter estimates of Inorganic Phosphate (Pi) levels , derived from participant cranial 31P-Magnetic Resonance Spectroscopy (MRS) centered on the basal ganglia and related motor regions. [Time Frame: Timepoint: 48 weeks (visit 5)]<br>Patients who consent to having the 31P-MR spectroscopy, data will be analysed for the change in energy metabolic levels at baseline and visit 5 (week 48).<br>• Mean change from baseline to week 48 in objective quantification of participant motor impairment, using motion sensors. [Time Frame: Timepoint: 48 weeks (visit 5)]<br>For the subset of patients who consent to having the Opticals sensor based gait assessment, the data will be analysed for changes in motor impairment at baseline and visit 5 (week 48).

For peer review only

**Brief Title**	Trial of Ursodeoxycholic Acid (UDCA) for Parkinson's Disease: The "UP" Study
**Official Title**	A Phase II, Placebo Controlled, Double Blind, Randomised Clinical Trial To Assess The Safety And Tolerability Of 30mg/kg Daily Ursodeoxycholic Acid (UDCA) In Patients With Parkinson's Disease (PD)
**Study Type**	Interventional
**Study Phase**	Phase 2
**Study Design**	Allocation: Randomized<br>Intervention Model: Parallel Assignment<br>Intervention Model Description:<br><br>A randomised double-blind, placebo controlled 48 week trial of UDCA at a daily dose of 30 mg/kg in patients with early Parkinson's disease <3 years post diagnosis.<br><br>Masking: Triple (Participant, Investigator, Outcomes Assessor)<br>Masking Description:<br>This is a double-blind trial. The investigators, clinical study team, participants and analysing statistician will be blind to treatment allocation. The active treatment will be over-encapsulated and a matched placebo manufactured to maintain the blind.<br><br>The Independent Data Monitoring Committee (IDMC) is the only oversight body that has access to unblinded accumulating comparative data.<br><br>Primary Purpose: Other
**Condition**	Parkinson's Disease
**Intervention**	Drug: Ursonorm<br>Ursodeoxycholic acid<br>Other Name: UDCA
**Study Arms**	• Placebo Comparator: Placebo<br>2:1 in favour of UDCA<br>Intervention: Drug: Ursonorm

For peer review only

	• Experimental: Ursonorm (Ursodeoxycholic acid)<br>UDCA 30 mg/kg daily, tablet form taking orally , administered 3 monthly for 12 months, dose titration during the 1st month will occur.<br>Intervention: Drug: Ursonorm
**Recruitment Status**	Recruiting
**Estimated Enrollment**	30
**Estimated Study Completion Date**	September 2020
**Eligibility Criteria**	Inclusion Criteria:<br>• Diagnosis of Parkinson's disease: PD is a clinical diagnosis as defined by the Queen Square Brain Bank criteria (bradykinesia defined as slowness of initiation of voluntary movement with progressive reduction in speed and amplitude on repetitive actions and at least one of the following: Rigidity, 4-6 Hz rest tremor). The diagnosis will have been made by the treating clinician and confirmed by the PI on site after review of the clinical history, examination findings and response to PD medication.<br>The Queen Square brain bank criteria MAY be used to help assist in the diagnosis although this need not be a formal inclusion criteria, and the relevance of a positive family history of PD, or a confirmed genetic basis for an individual's symptoms will be evaluated in the context of other clinical features in determining diagnosis and eligibility.<br>Diagnosis of Parkinson's disease ≤ 3 years ago by a clinician with particular expertise in the diagnosis and treatment of movement disorders (typically one of the PIs or their consultant colleagues). The date of diagnosis will be verified by a review of the medical records.

For peer review only

	Subjective improvement of motor impairment on dopaminergic medication, confirmed by PI through personal examination and/or review of medical records
	Hoehn and Yahr stage ≤ 2.5 in the practically defined "ON" medication state. This implies that all patients will be mobile without assistance during their best "ON" medication periods.
	Ability to take study drug
	Ability to communicate in English
	Age 18 - 75 yr of any gender
	Documented informed consent to participate
	Able to comply with study protocol and willing to attend necessary study visits
	Exclusion Criteria:
	Diagnosis or suspicion of other cause of parkinsonism such as Multiple system atrophy (MSA) or progressive supranuclear palsy (PSP), drug induced parkinsonism, dystonic tremor or essential tremor will not be recruited.
	Known abnormality on CT or MRI brain imaging considered likely to compromise compliance with trial/protocol/31P-MRS acquisition.
	Known claustrophobia or other reasons why patient could not tolerate or be suitable for 31P-MR Spectroscopy (31P-MRS)
	Current or previous exposure to UDCA
	Current or previous diagnosis of liver disease judged to be significant by the clinical investigator, in particular Primary Biliary Cholangitis (previously referred to as Primary Biliary Cirrhosis, PBC)
	Prior intracerebral surgical intervention for PD (including deep-brain stimulation). Patients who have previously undergone deep brain stimulation, intracerebral administration of growth factors, gene therapies or cell therapies will not be eligible.
	Already actively participating in a trial of a device, drug or surgical treatment for PD
	History of alcoholism
	Women of child - bearing potential (WOCBP)
	Participants who lack the capacity to give informed consent

For peer review only

	Any medical or psychiatric condition which in the investigator's opinion compromises the potential participant's ability to participate<br>Concurrent dementia defined by Montreal Cognitive assessment (MoCA) score <25<br>Concurrent severe depression defined by a score >16 on the Montgomery- Asberg Depression Rating Scale (MADRS)<br>Serum transaminases (such as aspartate transaminase (AST) more than 2 times upper limit of normal.<br>Patients on ciclosporin, nitrendipine or dapsone for the treatment of concomitant, general medical conditions.<br>Participants with previous or current diagnosis of inflammatory bowel disease (i.e. ulcerative colitis or Crohn's disease)		
**Sex/Gender**	Sexes Eligible for Study:	All	
**Ages**	18 Years to 75 Years (Adult, Older Adult)		
**Accepts Healthy Volunteers**	No		
**Contacts**	Contact: Sarah Moll<br>Contact: Jodie Keyworth	0114 2712563 ext 12563<br>0114 2265394 ext 65394	sarah.moll@sth.nhs.uk<br>jodie.keyworth@sth.nhs.uk
**Listed Location Countries**	United Kingdom		
**NCT Number**	NCT03840005		
**Other Study ID Numbers**	STH18493<br>2018-001887-46 (EudraCT Number)		
**IPD Sharing Statement**	Plan to Share IPD:<br>Plan Description:	Yes<br>The results of this trial will be submitted for publication in a peer reviewed journal, in addition to reports at appropriate specialist conferences. The results of the trial will be	

For peer review only

		disseminated regardless of the direction of effect. No participants will be identified during this process.
	Supporting Materials:	Study Protocol
	Time Frame:	Requests for the supporting information will be considered on a case by case basis with the CI and sponsor in conjunction with contract agreements with collaborators
	Access Criteria:	As above
**Study Sponsor**	Sheffield Teaching Hospitals NHS Foundation Trust	
**Collaborators**	• Prof Claudia Mazza and Dr Ellen Buckley, INSIGNEO, University of Sheffield<br>• PRO.MED.CS Praha a.s.	
**Investigators**	Principal Investigator:	Oliver Bandmann Sheffield Teaching Hospitals NHS Foundation Trust

For peer review only

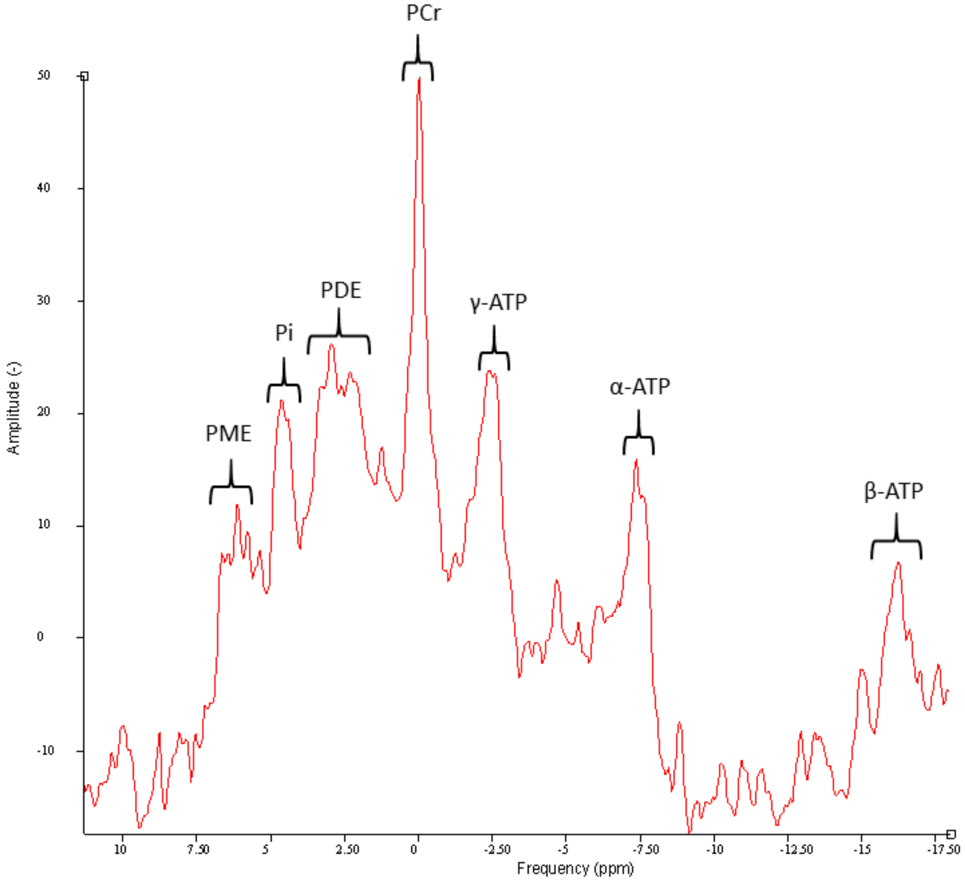

Figure 1: Representative 31P-MRS spectra obtained from the midbrain of a healthy volunteer following appropriate phasing and 10Hz Lorentzian apodization. From left to right, phosphomonoesters (PME), inorganic phosphate (Pi), phosphodiesters (PDE), phosphocreatine (PCr), and the three spectral resonances of adenosine triphosphate (γ-,α-,β-ATP).

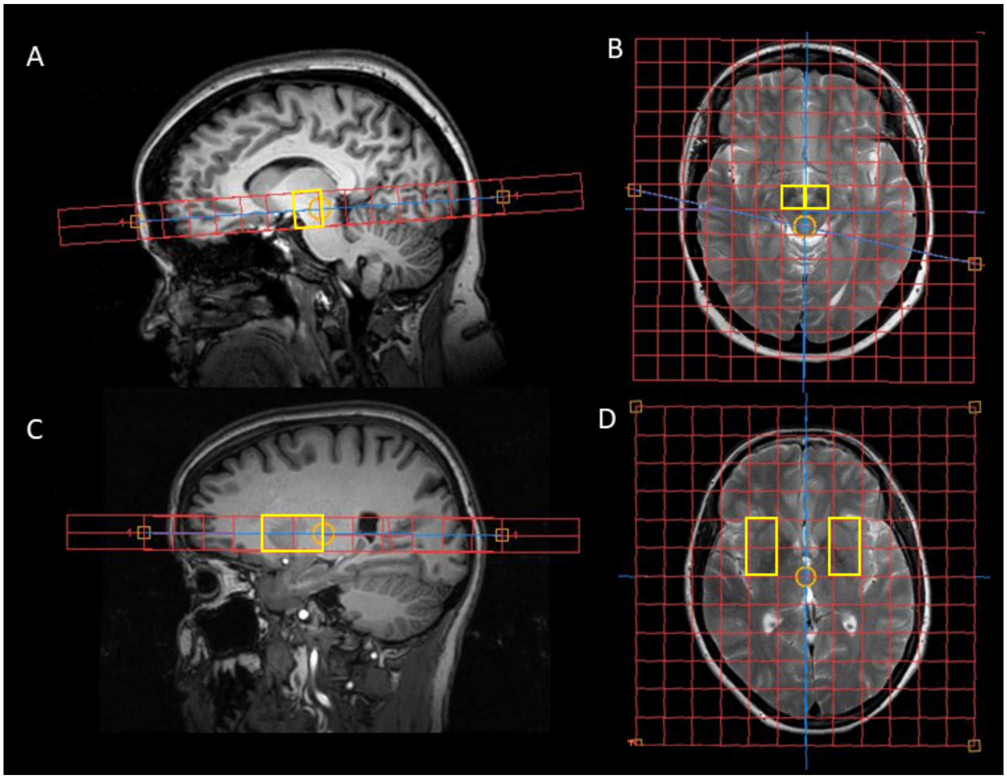

Figure 2: The substantia nigra slice is placed to cover the midbrain with the highlighted voxels of interest for subsequent analyses highlighted in yellow in the sagittal (A) and axial planes (B).Placement of 31P-MRS slices. The basal ganglia slice is placed over the putamen aligned in both the coronal (C) axial planes (D), and voxels of interest for subsequent analyses are highlighted in yellow. One voxel covers the anterior putamen and another the posterior putamen.

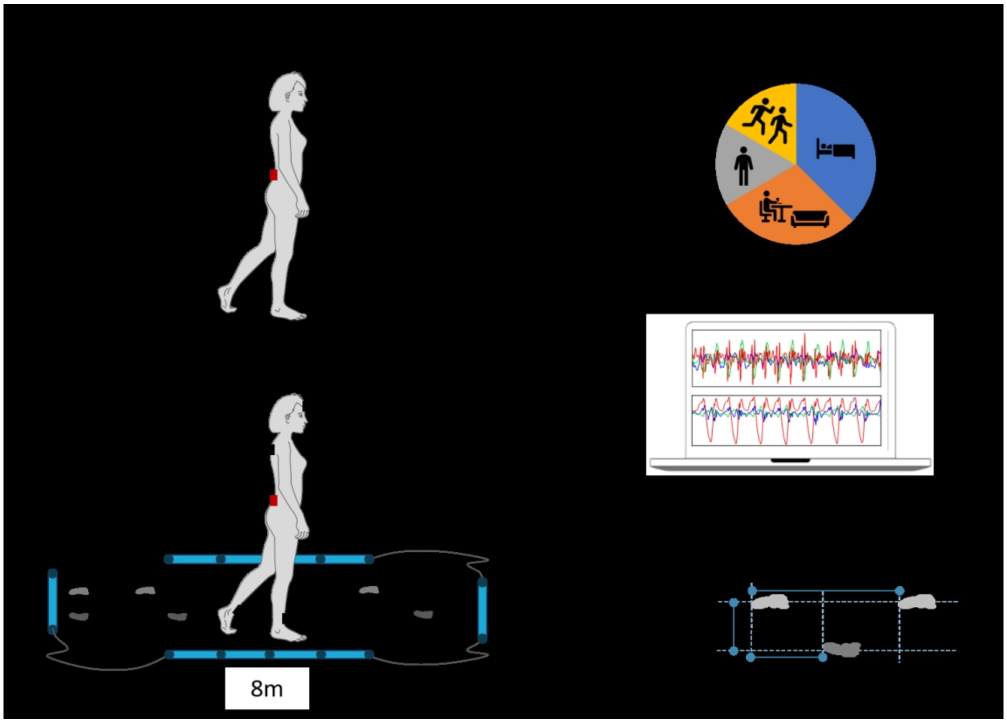

Figure 3: Protocols deployed at the two sites. All participants undergo seven day physical activity monitoring in order to estimate physical activity levels and capture temporal and gait quality measures in a real-world setting. In-clinic instrumented gait tasks are also completed at both sites to provide spatiotemporal and gait quality measures of gait capacity. At UCL only red sensor location is implemented.

249x178mm (150 x 150 DPI)

Reporting checklist for protocol of a clinical trial.

Based on the SPIRIT guidelines.

Instructions to authors

Complete this checklist by entering the page numbers from your manuscript where readers will find each of the items listed below.

Your article may not currently address all the items on the checklist. Please modify your text to include the missing information. If you are certain that an item does not apply, please write "n/a" and provide a short explanation.

Upload your completed checklist as an extra file when you submit to a journal.

In your methods section, say that you used the SPIRITreporting guidelines, and cite them as:

Chan A-W, Tetzlaff JM, Altman DG, Laupacis A, Gøtzsche PC, Krleža-Jerić K, Hróbjartsson A, Mann H, Dickersin K, Berlin J, Doré C, Parulekar W, Summerskill W, Groves T, Schulz K, Sox H, Rockhold FW, Rennie D, Moher D. SPIRIT 2013 Statement: Defining standard protocol items for clinical trials. Ann Intern Med. 2013;158(3):200-207

		Reporting Item	Page Number
**Administrative information**			
Title	#1	Descriptive title identifying the study design, population, interventions, and, if applicable, trial acronym	1
Trial registration	#2a	Trial identifier and registry name. If not yet registered, name of intended registry	3
Trial registration: data set	#2b	All items from the World Health Organization Trial Registration Data Set	36
Protocol version	#3	Date and version identifier	1
Funding	#4	Sources and types of financial, material, and other support	22
Roles and responsibilities: contributorship	#5a	Names, affiliations, and roles of protocol contributors	1, 22

For peer review only

Roles and responsibilities: sponsor contact information	#5b	Name and contact information for the trial sponsor	22
Roles and responsibilities: sponsor and funder	#5c	Role of study sponsor and funders, if any, in study design; collection, management, analysis, and interpretation of data; writing of the report; and the decision to submit the report for publication, including whether they will have ultimate authority over any of these activities	22
Roles and responsibilities: committees	#5d	Composition, roles, and responsibilities of the coordinating centre, steering committee, endpoint adjudication committee, data management team, and other individuals or groups overseeing the trial, if applicable (see Item 21a for data monitoring committee)	n/a
**Introduction**			
Background and rationale	#6a	Description of research question and justification for undertaking the trial, including summary of relevant studies (published and unpublished) examining benefits and harms for each intervention	5
Background and rationale: choice of comparators	#6b	Explanation for choice of comparators	8
Objectives	#7	Specific objectives or hypotheses	5
Trial design	#8	Description of trial design including type of trial (eg, parallel group, crossover, factorial, single group), allocation ratio, and framework (eg, superiority, equivalence, non-inferiority, exploratory)	7
**Methods: Participants, interventions, and outcomes**			
Study setting	#9	Description of study settings (eg, community clinic, academic hospital) and list of countries where data will be collected. Reference to where list of study sites can be obtained	7
Eligibility criteria	#10	Inclusion and exclusion criteria for participants. If applicable, eligibility criteria for study centres and individuals who will	7

For peer review only

		perform the interventions (eg, surgeons, psychotherapists)	
Interventions: description	#11a	Interventions for each group with sufficient detail to allow replication, including how and when they will be administered	10
Interventions: modifications	#11b	Criteria for discontinuing or modifying allocated interventions for a given trial participant (eg, drug dose change in response to harms, participant request, or improving / worsening disease)	13
Interventions: adherance	#11c	Strategies to improve adherence to intervention protocols, and any procedures for monitoring adherence (eg, drug tablet return; laboratory tests)	9, 13
Interventions: concomitant care	#11d	Relevant concomitant care and interventions that are permitted or prohibited during the trial	n/a
Outcomes	#12	Primary, secondary, and other outcomes, including the specific measurement variable (eg, systolic blood pressure), analysis metric (eg, change from baseline, final value, time to event), method of aggregation (eg, median, proportion), and time point for each outcome. Explanation of the clinical relevance of chosen efficacy and harm outcomes is strongly recommended	13-17
Participant timeline	#13	Time schedule of enrolment, interventions (including any run-ins and washouts), assessments, and visits for participants. A schematic diagram is highly recommended (see Figure)	10
Sample size	#14	Estimated number of participants needed to achieve study objectives and how it was determined, including clinical and statistical assumptions supporting any sample size calculations	11
Recruitment	#15	Strategies for achieving adequate participant enrolment to reach target sample size	7
**Methods: Assignment of interventions (for controlled trials)**			
Allocation: sequence generation	#16a	Method of generating the allocation sequence (eg, computer-generated random numbers), and list of any factors for stratification. To reduce predictability of a random sequence, details of any planned restriction (eg, blocking) should be provided in a separate document that is unavailable to those who enrol participants or assign interventions	9

For peer review only

Allocation concealment mechanism	#16b	Mechanism of implementing the allocation sequence (eg, central telephone; sequentially numbered, opaque, sealed envelopes), describing any steps to conceal the sequence until interventions are assigned	9
Allocation: implementation	#16c	Who will generate the allocation sequence, who will enrol participants, and who will assign participants to interventions	9
Blinding (masking)	#17a	Who will be blinded after assignment to interventions (eg, trial participants, care providers, outcome assessors, data analysts), and how	10
Blinding (masking): emergency unblinding	#17b	If blinded, circumstances under which unblinding is permissible, and procedure for revealing a participant's allocated intervention during the trial	13
**Methods: Data collection, management, and analysis**			
Data collection plan	#18a	Plans for assessment and collection of outcome, baseline, and other trial data, including any related processes to promote data quality (eg, duplicate measurements, training of assessors) and a description of study instruments (eg, questionnaires, laboratory tests) along with their reliability and validity, if known. Reference to where data collection forms can be found, if not in the protocol	13-17
Data collection plan: retention	#18b	Plans to promote participant retention and complete follow-up, including list of any outcome data to be collected for participants who discontinue or deviate from intervention protocols	17
Data management	#19	Plans for data entry, coding, security, and storage, including any related processes to promote data quality (eg, double data entry; range checks for data values). Reference to where details of data management procedures can be found, if not in the protocol	18
Statistics: outcomes	#20a	Statistical methods for analysing primary and secondary outcomes. Reference to where other details of the statistical analysis plan can be found, if not in the protocol	17
Statistics: additional analyses	#20b	Methods for any additional analyses (eg, subgroup and adjusted analyses)	17

For peer review only

Statistics: analysis population and missing data	#20c	Definition of analysis population relating to protocol non-adherence (eg, as randomised analysis), and any statistical methods to handle missing data (eg, multiple imputation)	17
**Methods: Monitoring**			
Data monitoring: formal committee	#21a	Composition of data monitoring committee (DMC); summary of its role and reporting structure; statement of whether it is independent from the sponsor and competing interests; and reference to where further details about its charter can be found, if not in the protocol. Alternatively, an explanation of why a DMC is not needed	13
Data monitoring: interim analysis	#21b	Description of any interim analyses and stopping guidelines, including who will have access to these interim results and make the final decision to terminate the trial	n/a
Harms	#22	Plans for collecting, assessing, reporting, and managing solicited and spontaneously reported adverse events and other unintended effects of trial interventions or trial conduct	13
Auditing	#23	Frequency and procedures for auditing trial conduct, if any, and whether the process will be independent from investigators and the sponsor	13
**Ethics and dissemination**			
Research ethics approval	#24	Plans for seeking research ethics committee / institutional review board (REC / IRB) approval	18
Protocol amendments	#25	Plans for communicating important protocol modifications (eg, changes to eligibility criteria, outcomes, analyses) to relevant parties (eg, investigators, REC / IRBs, trial participants, trial registries, journals, regulators)	18
Consent or assent	#26a	Who will obtain informed consent or assent from potential trial participants or authorised surrogates, and how (see Item 32)	8-9
Consent or assent: ancillary studies	#26b	Additional consent provisions for collection and use of participant data and biological specimens in ancillary studies, if applicable	n/a
Confidentiality	#27	How personal information about potential and enrolled participants will be collected, shared, and maintained in order to protect confidentiality before, during, and after the trial	18

For peer review only

Declaration of interests	#28	Financial and other competing interests for principal investigators for the overall trial and each study site	22
Data access	#29	Statement of who will have access to the final trial dataset, and disclosure of contractual agreements that limit such access for investigators	n/a
Ancillary and post trial care	#30	Provisions, if any, for ancillary and post-trial care, and for compensation to those who suffer harm from trial participation	n/a
Dissemination policy: trial results	#31a	Plans for investigators and sponsor to communicate trial results to participants, healthcare professionals, the public, and other relevant groups (eg, via publication, reporting in results databases, or other data sharing arrangements), including any publication restrictions	18
Dissemination policy: authorship	#31b	Authorship eligibility guidelines and any intended use of professional writers	22
Dissemination policy: reproducible research	#31c	Plans, if any, for granting public access to the full protocol, participant-level dataset, and statistical code	n/a
**Appendices**			
Informed consent materials	#32	Model consent form and other related documentation given to participants and authorised surrogates	n/a
Biological specimens	#33	Plans for collection, laboratory evaluation, and storage of biological specimens for genetic or molecular analysis in the current trial and for future use in ancillary studies, if applicable	n/a

The SPIRIT checklist is distributed under the terms of the Creative Commons Attribution License CC-BY-ND 3.0. This checklist was completed on 24. March 2020 using https://www.goodreports.org/, a tool made by the EQUATOR Network in collaboration with Penelope.ai

For peer review only

BMJ Open

The UP Study – Ursodeoxycholic acid as a novel disease-modifying treatment for Parkinson's disease: Protocol for a two-centre, randomized, double-blind, placebo-controlled trial

Journal:	*BMJ Open*
Manuscript ID	bmjopen-2020-038911.R1
Article Type:	Protocol
Date Submitted by the Author:	28-May-2020
Complete List of Authors:	Payne, Thomas; The University of Sheffield Institute for Translational Neuroscience; NIHR Sheffield Biomedical Research Centre<br>Sassani, Matilde; The University of Sheffield Institute for Translational Neuroscience<br>Buckley, Ellen; NIHR Sheffield Biomedical Research Centre; The University of Sheffield, Institute for In Silico Medicine<br>Moll, Sarah; NIHR Sheffield Biomedical Research Centre<br>Anton, Adriana; NIHR Sheffield Biomedical Research Centre; The University of Sheffield, Academic Unit of Radiology<br>Appleby, Matthew; University College London Institute of Neurology<br>Maru, Seema; University College London Institute of Neurology<br>Taylor, Rosie; The University of Sheffield, Statistical Services Unit<br>McNeill, Alisdair; The University of Sheffield Institute for Translational Neuroscience<br>Hoggard, N; The University of Sheffield, Academic Unit of Radiology<br>Mazza, Claudia; The University of Sheffield, Institute for In Silico Medicine<br>Wilkinson, Iain; The University of Sheffield, Academic Unit of Radiology<br>Jenkins, Thomas; The University of Sheffield Institute for Translational Neuroscience<br>Foltynie, Thomas; University College London Institute of Neurology<br>Bandmann, O; The University of Sheffield Institute for Translational Neuroscience; NIHR Sheffield Biomedical Research Centre
**Primary Subject Heading**:	Neurology
Secondary Subject Heading:	Pharmacology and therapeutics, Radiology and imaging
Keywords:	Parkinson-s disease < NEUROLOGY, Neurology < INTERNAL MEDICINE, Magnetic resonance imaging < RADIOLOGY & IMAGING, Clinical trials < THERAPEUTICS, Adult neurology < NEUROLOGY, Neuroradiology < NEUROLOGY

SCHOLARONE™ Manuscripts

I, the Submitting Author has the right to grant and does grant on behalf of all authors of the Work (as defined in the below author licence), an exclusive licence and/or a non-exclusive licence for contributions from authors who are: i) UK Crown employees; ii) where BMJ has agreed a CC-BY licence shall apply, and/or iii) in accordance with the terms applicable for US Federal Government officers or employees acting as part of their official duties; on a worldwide, perpetual, irrevocable, royalty-free basis to BMJ Publishing Group Ltd ("BMJ") its licensees and where the relevant Journal is co-owned by BMJ to the co-owners of the Journal, to publish the Work in this journal and any other BMJ products and to exploit all rights, as set out in our licence.

The Submitting Author accepts and understands that any supply made under these terms is made by BMJ to the Submitting Author unless you are acting as an employee on behalf of your employer or a postgraduate student of an affiliated institution which is paying any applicable article publishing charge ("APC") for Open Access articles. Where the Submitting Author wishes to make the Work available on an Open Access basis (and intends to pay the relevant APC), the terms of reuse of such Open Access shall be governed by a Creative Commons licence – details of these licences and which Creative Commons licence will apply to this Work are set out in our licence referred to above.

Other than as permitted in any relevant BMJ Author's Self Archiving Policies, I confirm this Work has not been accepted for publication elsewhere, is not being considered for publication elsewhere and does not duplicate material already published. I confirm all authors consent to publication of this Work and authorise the granting of this licence.

For peer review only

The UP Study –

Ursodeoxycholic acid as a novel disease-modifying treatment for Parkinson's disease: Protocol for a two-centre, randomized, double-blind, placebo-controlled trial

T. Payne[1,2], M. Sassani[1], E. Buckley[2,3], S. Moll[2], A. Anton[2,4], M. Appleby[5], S. Maru[5], R. Taylor[6], A.McNeill[1,2,3], N. Hoggard[4], C. Mazzà[2,3]; I.D. Wilkinson[2,4], T. Jenkins[1,2], T. Foltynie[5], O. Bandmann[1,2]

[1]Sheffield Institute for Translational Neuroscience (SITraN), Dept Neuroscience, University of Sheffield, Sheffield, UK; [2]National Institute for Health Research, Biomedical Research Centre, Royal Hallamshire Hospital, Sheffield, UK, [3]Insigneo Institute for In Silico Medicine, University of Sheffield, Sheffield, UK; [4]Academic Unit of Radiology, University of Sheffield, Sheffield, UK; [5]Institute of Neurology, UCL, London, UK; [6]Statistical Services Unit, University of Sheffield, Sheffield, UK

Corresponding Author: Prof Oliver Bandmann, Sheffield Institute for Translational Neuroscience, Unviersity of Sheffield, 385a Glossop Road, Sheffield, S10 2HQ.

o.bandmann@sheffield.ac.uk

Word count: 3,933

Version: 1.0 24/3/2020

For peer review only

Abstract

Introduction: There are no disease modifying treatments for Parkinson's Disease (PD). We undertook the first drug screen in PD patient tissue and identified Ursodeoxycholic acid (UDCA) as a promising mitochondrial rescue agent. The aims of this trial are to determine safety and tolerability of UDCA in PD at 30mg/kg, confirm the target engagement of UDCA, apply a novel motion-sensor based approach to quantify disease progression objectively, and estimate the mean effect size and its variance on the change in motor severity.

Methods and Analysis: This is a phase II, two-centre, double-blind, randomised, placebo-controlled trial of UDCA at a dose of 30mg/kg in 30 participants with early PD. Treatment duration is 48 weeks, followed by an 8 week washout phase. Randomisation is 2:1, drug to placebo. Assessments are performed at baseline, week 12, 24, 36, 48 and 56. The primary outcome is safety and tolerability. Secondary outcomes will compare the change between baseline and week 48 using the following three approaches: the Movement Disorders Society Unified Parkinson's Disease Rating Scale Part III in the practically defined 'OFF' medication state; confirmation of target engagement, applying ^{31}Phosphorus Magnetic Resonance Spectroscopy to assess the levels of ATP and relevant metabolites in the brain; and objective quantification of motor impairment, using a validated, motion-sensor based approach. The primary outcome will be reported using descriptive statistics and comparisons between treatment groups. For each secondary outcome the change from baseline will be summarised within treatment groups using summary statistics and appropriate statistical tests assessing for significant differences. All outcomes will use an intention-to-treat analysis population.

For peer review only

Ethics and Dissemination: This trial has been approved by the East of England – Cambridgeshire and Hertfordshire Research Ethics committee. Results will be disseminated in peer-reviewed journals, presentations at scientific meetings and to patients in a lay-summary format.

Trial registration: ClinicalTrials.gov: NCT03840005

Strengths and limitations of this study

- This is the first double-blind, randomised, placebo-controlled trial of Ursodeoxycholic Acid (UDCA) in Parkinson's Disease (PD).
- This study uses novel secondary outcomes not previously used in a clinical trial studying PD; namely ^{31}Phosphorus Magnetic Resonance Spectroscopy of disease specific regions and detailed, complementary home and clinic-based motor activity and gait analysis.
- ^{31}P-MRS will all the assessment of mitochondrial dysfunction directly in the substantia nigra, the most severely affected brain area in PD.
- A limitation of the study is the considerable number of capsules patients will have to take; patients will on average be taking an additional nine extra capsules of medication each day through the trial, significantly increasing their 'pill burden'.
- A further limitation is the small sample size of n=30 with 20 patients on UDCA and 10 patients on placebo, it will not be possible to draw firm conclusions about the neuroprotective effect of UDCA in PD but will allow for appropriate power and sample size calculations for future studies.

For peer review only

INTRODUCTION

Parkinson's Disease (PD) is a progressive neurodegenerative disorder comprising gait impairment, bradykinesia, rigidity and tremor[1]. It is the second most common neurodegenerative disorder predicted to double in global prevalence between 2005 and 2030[2]. Developing disease modifying therapies is a crucial step in reducing the associated morbidity of PD and to delay the development of late stage complications such as dementia, postural instability and psychosis.

Mitochondrial dysfunction is a key pathogenic mechanism in both sporadic and familial PD and therefore a promising target for disease-modifying therapy[3]. Our group undertook the first drug screen in genetically stratified PD patient tissue[4 5]. This approach identified ursodeoxycholic acid (UDCA) as a particularly promising mitochondrial rescue compound[5]. Other groups demonstrated independently the neuroprotective effect of UDCA and its taurine conjugate TUDCA in the 1-methyl-4-phenyl-1,2,3,6-tetrahydropyridine (MPTP) mouse model and the rotenone rat model of PD[6 7].

The mode of action of UDCA remains to be fully elucidated. Current literature would suggest that it appears to be Akt mediated. Both Ursocholanic acid and TUDCA have been demonstrated to induce Akt phosphorylation[4 7]. Akt activation requires phosphorylation at two sites and promotes cell survival through several mechanisms, failure of activation is a common finding underlying neurodegeneration[4]. Reduced Akt signalling has been found in in-vitro models of PD and in sporadic PD brains post-mortem in the substantia nigra[8 9].

UDCA has been in clinical use for decades primarily for primary biliary cholangitis (previously primary biliary cirrhosis) with excellent safety and tolerability at the standard dose of 15mg/kg[10]. UDCA has also been well tolerated at a higher dose of

For peer review only

30 mg/kg over two years in patients with primary sclerosing cholangitis[11]. UDCA is a naturally occurring bile acid but normally only forms 1-3% of total endogenous human bile acids. However, in patients on standard therapeutic doses of UDCA (13-15 mg/kg/day), UDCA may form up to 40% of total bile acids. Intestinal absorption after an oral dose is high with a first-pass clearance of about 50-60%. Plasma levels reach maximum concentrations after 60 minutes after ingestion with another peak at 3 hours[12].

A pharmacokinetic study of UDCA in Motor Neuron Disease demonstrated a significant correlation between serum concentration at one hour post dose and CSF concentration two hours post dose, with most of the variability in CSF concentrations (78%) explained by variability in serum concentrations. Mean CSF concentration post-dose at 15mg/kg was 86.69nmol/L, at 30mg/kg was 114.22nmol/L and 50mg/kg was 191.11 nmol/L[13].

The main objectives of this trial (The UP Study) are to demonstrate the safety and tolerability of UDCA in PD at a dose of 30mg/kg and to explore the effects of UDCA on novel outcome measures such as ^{31}Phosphorus Magnetic Resonance Spectroscopy (^{31}P-MRS) and the objective quantification of motor impairment, using a sensor-based approach. Additionally, we hope to collect an estimate of the effect size and variance of UDCA on the change in motor severity of PD over 1 year compared to placebo using long-established clinical assessment tools.

For peer review only

METHODS AND ANALYSIS

Design

This is a phase II, two-centre, double-blind, randomised, placebo-controlled trial of 30mg/kg in Ursodeoxycholic acid in early PD. Treatment duration with drug or placebo is 48 weeks in total, followed by an 8 week washout phase. 30 participants will be included. Randomisation is 2:1 in favour of drug to placebo. The choice of 30mg/kg day has been informed by previous pharmacokinetic studies in Motor Neuron Disease, this dose allows effective penetrance of the CNS but also balances the exposure to a potentially higher risk of side effects with increasing doses and possible issues with compliance due to the then very large number of additional tablets the patients would need to take[13].

Participants

Patients with early PD, as defined by a clinical diagnosis made by a Movement Disorders Specialist according to the Queen Square Brain Bank Criteria within 3 years prior to recruitment and who demonstrate a clear subjective response to dopaminergic medication, confirmed by the treating physician, will be recruited from two sites; Sheffield Teaching Hospitals NHS Trust (STH) and University College London Hospitals NHS Foundation Trust (UCLH). Key inclusion and exclusion criteria can be found in Table 1[14].

Participants are typically recruited through specialist Movement Disorders Clinics at both trial sites. The trial has also been advertised online by the Parkinson's UK website, the Cure Parkinson's Trust, the Sheffield National Institute for Health-Related Research (NIHR)-Biomedical Research Centre website (NIHR-BRC) and the NIHR Clinical Research Network websites. Trial advertisements direct participants to contact

For peer review only

the STH study team to be provided with a Patient Information Sheet (PIS) and a reply slip to confirm ongoing interest and to organise a pre-screening telephone call to confirm eligibility and suitability for the study.

Study visits either take place at the Clinical Research Facility (CRF) of the Royal Hallamshire Hospital, Sheffield, for STH participants or at the Leonard Wolfson Experimental Neurology Centre, Queen Square, London for UCLH participants.

Primary Outcome

The primary outcome for the UP study is to compare the safety and tolerability of UDCA at 30 mg/kg in PD compared to placebo as indicated by the following: the number of serious adverse events (SAEs), number of adverse treatment-reactions and the number of patients completing the study. The safety and tolerability of UDCA in this study will be compared descriptively with the reported safety and tolerability of Exenatide in the Exenatide-PD trial which followed a broadly similar trial design[15].

Secondary Outcomes

The effect of UDCA versus placebo will be assessed as a change from baseline to week 48 for the following secondary outcomes:

1. Clinical assessment using the Movement Disorders Society Unified Parkinson's Disease Rating Scale (MDS-UPDRS) Part 3 motor examination in the practically-defined "OFF" medication state.

2. *In-vivo* measures of high and low energy metabolite levels (including ATP, phosphocreatine and inorganic phosphate) derived from multi-voxel brain ^{31}P-MRS at baseline and week 48.

3. Sensor-based, objective quantification of motor impairment using data collected with wearable sensors both in supervised (OptoGait and Opals systems, Sheffield

patients only, Dynaport Movemonitor+, all patients) as well as in unsupervised real-life conditions (Dynaport Movemonitor+, all patients).

Screening Visit

Participants likely to be eligible will be invited for a screening visit where all inclusion and exclusion criteria will be reviewed. Participants will be offered the opportunity to discuss the trial and have all questions answered after which they will be asked to provide written informed consent before proceeding to further assessment. Participants will have a full demographic, medical and concomitant medication history taken and reviewed. A physical examination to confirm the diagnosis of PD and exclude PD ‘mimic’ conditions will be performed. A Montreal Cognitive Assessment (MoCA) and Montgomery-Asberg Depression Rating Scale (MADRS) will be performed to exclude concurrent dementia or severe active depression [16 17]. Safety bloods (full blood count, urea & electrolytes, liver function tests, blood glucose, HbA1C, lipid profile) and an ECG will be performed at the screening visit. If the participant remains eligible, they will be provided with an activity monitor (McRoberts, Dynaport MoveMonitor+) to wear for 1 week prior to the baseline visit as described later. For those undergoing ^{31}P-MRS, this will be arranged within 1 week before or on the day of the baseline visit, as described later. The baseline visit will be completed within 8 weeks of screening.

Baseline Visit, Randomisation and blinding

Randomisation to either active compound or placebo will be administered using a centralised, web-based system hosted by epiGenesys (a wholly owned subsidiary of

For peer review only - http://bmjopen.bmj.com/site/about/guidelines.xhtml

the University of Sheffield) on behalf of the University of Sheffield Clinical Trials Research Unit (CTRU).

MDS-UPDRS Part 3 Motor Examination is performed in the 'OFF' state[18]. The practically defined 'OFF' state in this study requires participants to not have taken medication for 8 hours in the case of any drug containing Levodopa, or at least 36 hours in the case of longer acting agents such as dopamine agonists or enzyme inhibitors.

The supervised gait analysis is performed using a combination of an instrumented photoelectric walkway system (Microgate, OptoGait) and inertial sensors (APDM, Opal) system as described below.

Participants will then be invited to take their usual dopaminergic medication and after a minimum of 60 minutes undergo the following procedures to reassess them in the practically defined ON: MDS-UPDRS Parts 1-4 I in the 'ON' state, Non-motor Symptom Questionnaire (NMS-QUEST) and The 39-Item Parkinson's Disease Questionnaire (PDQ-39)[18-20].

Intervention

All study medication is provided as a white powder in a hard clear gelatine capsule. Placebo and study drug are completely matched with no identifiable differences in taste, appearance or smell. All packaging and labelling is identical. Each capsule of the active drug contains 250mg of UDCA.

Treatment with UDCA is started at a dose of 250mg (one capsule) per day with an increase by 250mg every 3 days until the target dose is reached, which is divided into 3 doses[21]. Most patients are expected to reach their target dose within 3-4 weeks and be on 9-10 capsules per day.

For peer review only

All participants, trial management and medical staff will be blinded to treatment. Participants undergo clinical assessments by the same blinded assessor at each site who is not involved with safety, adverse event (AE) monitoring or dose titration to avoid any assessment bias or accidental unblinding.

Assessment procedures

Following randomisation, a total of 5 further visits will be completed at week 12, 24, 36, 48 and 56. At week 48, treatment is completed and all medication returned. A final visit at week 56 for final safety monitoring and outcome measurement completes the study. Week 12 and 36 are purely for safety monitoring and medication supply.

The MDS-UPDRS Part 3 is completed in the practically defined ‘ON’ state at week 24 and in the ‘OFF’ state at week 48 and 56. The complete MDS-UPDRS (Parts 1-4) is completed in the ‘ON’ state at baseline, week 48 and 56.

The ^{31}P-MRS is repeated in the 7 days prior to week 48 for UCLH participants and on the day of the week 48 visit for STH participants. The week-long unsupervised at-home physical activity monitoring is repeated in the 7 days prior to week 48.

The MoCA, NMS-QUEST, PDQ-39 and MADRS are repeated at week 48 and 56.

At each visit, safety bloods (full blood count, urea & electrolytes, liver function tests, blood glucose, HbA1C, lipid profile) will be obtained. In addition, at each visit a 20ml serum sample is taken for long term storage and future research. At the baseline visit, blood is taken for genetic analysis, this will be performed using the NeuroChip Assay that assesses for approximately 180,000 genetic variants associated with neurological diseases[22].

A full schedule of activities can be seen in Table 2.

For peer review only

Exploratory Outcomes

The exploratory outcomes will consist of the change between week 48 and 56 in the following: MDS-UPDRS part 3 'OFF' scores, complete MDS-UPDRS (parts 1-4) 'ON' scores, total Levodopa equivalent daily dose, MoCA, MADRS, NMS-QUEST and PDQ-39.

The repeat assessments at week 56 (8 weeks after cessation of the study medication) will help to determine whether there is a sustained effect of UDCA on both motor and non-motor aspects of PD which would be in keeping with the assumption of a neuroprotective effect. Conversely, a rapid deterioration of these clinical parameters after cessation of the study drug would suggest a symptomatic effect of UDCA.

As an additional variable to be used in exploratory analysis a validated prognostic model calculating the risk of progression to an unfavourable outcome (either postural instability or dementia at 5 years) will be applied to each participant[23]. We hope that this variable will account for some of the inherent heterogeneity among participants for their speed of clinical progression.

Sample Size

The primary outcome of interest for this study is the safety and tolerability of UDCA which will be assessed by comparing the rate of Serious Adverse Events (SAEs) in the UDCA and placebo groups, alongside review of adverse treatment reactions and study completion. As the study is a pilot, it is not powered to compare the SAE rate between the groups statistically, but any SAEs in either group will be presented descriptively, the placebo group providing a baseline against which to view any SAEs in the UDCA group. Should this study result in no SAEs then it would be of interest to determine how likely it is that a larger study would find an intolerable rate of SAEs. For

For peer review only

this purpose, we will consider the rate of SAEs reported in the Exenatide PD trial to be tolerable and acceptable (i.e. 20%)[15]. In this study, should no SAEs be found in the group receiving UDCA (n=20) then the likelihood that the true SAE rate is less than 20% is 0.990778.

The sample size has not been prospectively adjusted to account for any loss to follow-up. Instead, as the trial is of a relatively short duration we have instead allowed for any participants withdrawing from the study or lost to follow-up before the completion of 12 weeks of treatment to be replaced with a new participant.

The study has not been powered formally for the secondary or exploratory outcome measures, therefore interpretation will concentrate on observed trends and confidence intervals for estimated differences. The data collected for the secondary and exploratory outcomes will allow the estimation of the effect size and variance in each outcome to facilitate formal power calculations for future Phase III studies. Of note, there is currently no data using either ^{31}P-MRS or our sensor based approached quantification of motor impairment. The collection of such data is critical to allow high quality future trial design using these novel outcome measures.

Patient and Public Involvement

Patient representatives have been involved in the design of the study protocol and have contributed to the generation of participant facing study documentation. Recruitment to the study will be aided by both local PD groups and publicised by The Cure Parkinson's Trust, Parkinson's UK and Michael J Fox Foundation. Results will be disseminated to all participants upon completion of the trial.

For peer review only

OUTCOME MEASURES

Safety Monitoring

At each visit, participants are asked to report any adverse events that have occurred since the previous visit. AEs may also be detected by the study team reviewing the patient or through notification by the participant's primary care physician. All AEs are assessed by a study doctor for their severity, likely relationship to study drug and required action by a study doctor not involved in the blinded assessment of the patient. All SAEs will be recorded and reported to the sponsor regardless of relation to trial treatment within 24 hours. Any suspected unexpected serious adverse reactions (SUSARs) will be reported to the sponsor immediately to allow facilitation of unblinding as necessary. All AEs reported will be reviewed by the Trial Management Group (TMG), Trial Steering Group (TSG) and monitored by an Independent Data Monitoring Committee (IDMC).

Unblinding requests from other clinicians responsible for a patient's care will be handled by the Principal Investigator (PI) at each site. The PI at each site may also choose to unblind in response to reported AEs as they are reported.

In the event that side effects such as diarrhoea do not resolve and become persistent or intolerable then the patient can have their dose adjusted to their last tolerated dose for the remainder of the study.

All participants will be asked to return unused medication, this medication will be counted and recorded to assess compliance.

Motor Measures

The MDS-UPDRS, is currently the most utilised and validated clinical tool to quantify the disease state of an individual with PD[18]. The minimal clinically important difference

For peer review only

in the MDS-UPDRS Part 3 is reported to be an improvement of 3.25 points for detecting minimal, but clinically pertinent, improvement and a deterioration of 4.63 points for observing minimal, but clinically pertinent, worsening[24]. Over a period of 5 years MDS-UPDRS Part III scores were observed to increase (deteriorate) by 2.4 points per year[25]. However, despite expected annual deterioration being well characterised, rate of decline may still depend on disease stage and therefore contemporaneous placebo control data remains essential to evaluate potential new therapies.

Neuropsychological Measures

The MoCA is a globally used and validated measure of cognitive impairment and has been used a broad range of neurological diseases and study designs[16]. The MADRS has been validated in PD as a screening tool for major depression[17 26].

Non-motor and Quality of Life Measures

NMS-QUEST is a clinical screening tool that covers a wide range of non-motor symptoms[20]. PDQ-39 is a validated and widely used quality of life questionnaire that covers a range of measures such as emotional wellbeing, activities of daily living and mobility in the context of PD[19].The total equivalent levodopa dose is calculated using calculations and equivalencies generated previously in a systematic review and allows quantitative comparisons between patients on different medication regimes[27].

^{31}Phosphorous Magnetic Resonance Spectroscopy

^{31}P-MRS is experienced by the patient in the same manner as a standard clinical MRI scan. As the metabolites of interest are phosphorus based, it provides the opportunity

For peer review only

to investigate key metabolites in bioenergetics such as ATP, phosphocreatine (PCr) and inorganic phosphate (Pi) which all have clear spectroscopic resonances (Figure 1). It is, therefore, an ideal approach to assess mitochondrial function *in-vivo*. Ratio measures such as Pi/ATP and PCr/ATP have been shown to reflect the status of different aspects of oxidative phosphorylation pathways[28].

Two-dimensional Chemical Shift Imaging (CSI) with Image-selected *in vivo* Spectroscopy (ISIS) will be used for spectral spatial localisation[29 30], with a dedicated multi-nuclear MRI system (Ingenia 3.0T, Philips Healthcare, Best, NL) and dual-tuned $^{1}H/^{31}P$ head coil (Rapid Biomedical, Würzburg, Germany). Standard clinical T1 and T2 weighted imaging will allow the alignment of the two ^{31}P axial CSI sequences as shown in Figure 2. The two sequences will be aligned to obtain spectra from both the putamen (voxels for both anterior and posterior putamen bilaterally) and the midbrain (one voxel for each left and right). This is a clear advantage over alternative techniques that typically utilise surface coils as it allows the localisation of spectra to these specific brain regions typically involved in early PD. Imaging both anatomical regions is of importance as one mechanism of mitochondrial dysfunction in PD may be that of retrograde axonal degeneration, therefore spectra from the striatum may show clear mitochondrial dysfunction even in early disease independent of findings in the midbrain. Previous cross-sectional work using a similar ^{31}P-MRS protocol has demonstrated reductions in ATP and PCr in PD compared to controls in both the putamen and midbrain[31]. Additionally, a further study demonstrated that Pi/ATP ratios were increased in PD compared to controls[32].

Details of the acquisition sequences are shown in Table 3. Spectra will be processed in the time domain using jMRUI software v5.2 (http://www.jmrui.eu) and the AMARES algorithm is used to determine the relative area under each peak[33-35]. Analysis of the

For peer review only

^{31}P-MRS data will focus on the change between randomisation and week 48 of normalised amplitudes of ATP, PCr and Pi, and ratio values such as PCr/ATP and Pi/ATP that assess bioenergetic dysfunction. All STH patients will undergo ^{31}P-MRS. UCLH patients are also invited to attend the STH site for ^{31}P-MRS.

Gait Analysis and Activity Monitoring

Physical activity and gait capacity will be assessed at two time points, namely prior to/during the baseline visit and prior to/during the week 48 visit at the end of the treatment period.

Physical activity will be assessed using home-based "real-life" monitoring for seven consecutive days. A lightweight physical activity monitor (PAM) containing a triaxial accelerometer, gyroscope, digital memory card and a battery (McRoberts, Dynaport Movemonitor+,Netherlands) has been selected for continuous monitoring in all participants. Participants will wear the device for seven consecutive days and complete a diary to quantify their physical activity and gait characteristics within their normal weekly routine in a "real-world" setting.

Gait capacity will be assessed during the study visits (Figure 3) using a combination of wearable inertial sensors and an instrumented walkway. In particular, participants will complete gait analysis tasks during baseline and week 48 at the respective centre's Clinical Research Facilities (STH and UCLH). Patients will complete three short gait tasks. First, participants will be asked to complete the 3m Timed Up and Go test walk at self-selected speed. It is an assessment of functional mobility that incorporates transitional actions of standing, turning, and sitting[36 37]. Then participants will complete two continuous gait tasks at self-selected preferred, and fast paced walking speeds. Each trial will consist of walking back and forth at least six times along the 8m walkway with periods of quiet standing recorded at the start and end of each

For peer review only

trial. At both sites, participants will wear the Dynaport Movemonitor+ during instrumented gait tasks. At the Sheffield site, an instrumented 8m walkway (OptoGait, Microgate Corporation, Bolzano, Italy) and a set of inertial sensors (Opals, APDM Inc, Portland, OR, USA) will also be implemented. The instrumented walkway uses bar-mounted LEDs in a two dimensional configuration. The infrared signals transmitted are broken by the movement of the research subject's feet during walking, and various spatiotemporal gait parameters such as step time, stride length, step width and stance time are computed. The system has a spatial resolution of 1cm and a temporal resolution of milliseconds. The data from the inertial sensors will be used to monitor truncal sway during walking and provide a set of additional digitally mobility outcomes associated to the quality of gait (e.g. gait smoothness, variability, symmetry, etc.) [38 39]. The sensors will be positioned at both ankles, the lower back (L5), upper back (C7) and forehead. Each sensor contains an accelerometer, gyroscope and magnetometer and records synchronised data wirelessly. Data will be analysed with previously published, validated state of the art algorithms, implemented in Matlab [38 40 41].

STATISTICAL ANALYSIS

These analyses will include all randomised patients (an intention to treat (ITT) analysis population). The Primary Outcome of safety and tolerability will be reported using descriptive statistics and comparisons between treatment groups. Demographic and clinical assessment data will be summarised.

For each of the secondary outcomes the change from baseline will be summarised within treatment groups using standard summary statistics (number of participants, mean, standard deviation, median, minimum and maximum) with appropriate

For peer review only

statistical tests assessing for significant differences depending upon the distribution of the data and any relevant co-variates.

DATA MANAGEMENT

Data will be kept in accordance with God Clinical Practice, the Data Protection Act 2018 and General Data Protection Regulations. Data management will be provided by the University of Sheffield Clinical Trials Research Unit (CTRU). All data will be entered remotely on to a centralised database held within the CTRU (Prospect) by a research study member at the study site. Access to Prospect is controlled by usernames and encrypted passwords.

All participants will be assigned a unique participant ID number at screening that will link all of the clinical information held about them on the study database. It will also be used in all correspondence between CTRU and participating centres.

ETHICS AND DISSEMINATION

This trial has been approved by the East of England – Cambridgeshire and Hertford Shire Research Ethics committee (Protocol ID: 18/EE/0280) in November 2018. The trial has been registered on ClinicalTrials.gov (ID: NCT03840005). The study will be conducted in accordance with the local R&D approval and the Declaration of Helsinki. All participants provide written informed consent prior to any study procedures commencing. The results will be published in a peer reviewed journal and presented at regional, national and international scientific meetings as appropriate. A plain English summary of the study results will be sent to the study participants once data analysis has been completed. Results of the study may also be presented at meetings of PD support groups or to other relevant lay audiences.

For peer review only

For peer review only

DISCUSSION

We propose a novel study design for early, proof of concept PD neuroprotection trials, combining assessment for safety and tolerability with ^{31}P-MRS-based assessment of target engagement of bioenergetics pathways and motion-sensor based objective quantification of disease progression. Our study protocol will be particularly powerful for any compound aiming to directly improve mitochondrial function in PD. Additionally, our approach of using ^{31}P-MRS also holds promise to determine biologically relevant target engagement for compounds aiming at genetically defined upstream targets such as antisense oligonucleotides (ASO) for *LRRK2* or antibody therapy for alpha-synuclein. Mitochondrial dysfunction is a well-recognized aspect of both LRRK2- and alpha-synuclein-associated PD[42 43].

A recent open-label study of UDCA over 6 weeks with an escalating dose up to 50mg/kg in 5 patients with mild to moderate PD found reasonable tolerability and also used ^{31}P-MRS to assess target engagement[44]. However, their ^{31}P-MRS imaging data was obtained in only 3 participants and their methodology differed in that a surface coil was used and to acquire occipital lobe spectra only.

In-depth sensor-based gait analysis has the potential to overcome the current limitations of the MDS-UPDRS-based clinical assessment[18]. Gait analysis provides a method of quantifying gait disability and postural instability and therefore has potential as an objective motor endpoint for future studies. There is clear evidence that greater axial involvement predicts a poorer outcome in PD with regard to both cognitive decline and postural instability[23]. It is therefore likely that the greatest value in sensor-based analysis is in assessing a combination of spatiotemporal and upper body gait characteristics both in the formal clinical setting but also in exploring real-life mobility through at-home monitoring[38 45 46].

For peer review only

UDCA has previously been trialled in another neurodegenerative disorder, motor neuron disease (MND) at doses of 15, 30 and 50 mg/kg in a total of 18 patients. Patients were treated for 4 weeks. The main adverse events were minor gastrointestinal side effects, graded as mild to moderate. Side effect profiles and frequency were broadly similar between groups without a clear dose correlation[13]. This represents grounds to hypothesise that the primary outcome of safety and tolerability of UDCA at 30 mg/kg in PD will be achievable. We expect completion of the study analysis by July 2021.

For peer review only

Author Contributions

OB is responsible for the overall trial design with contributions from TF. SM led the overall administration and preparation of the trial. TF, S. Maru and MA deliver the trial at the UCLH site. TP, MS, AA, NH, IDW and TJ are responsible for the implementation and analysis of the ^{31}P-MRS. EB, AM and CM are responsible for the implementation and analysis of the sensor-based movement analysis tools. RT is responsible for statistical support of the trial and the power calculations provided. TP and EB are responsible for preparing the manuscript under the supervision of OB. All authors have reviewed and commented on this paper. The sponsor has reviewed all participant-facing documents as part of the ethics application (contact Sarah Moll, sarah.moll2@nhs.net, 0114 2712563).There are no competing interests declared by any author.

Funding Statement

This research was co-funded by the NIHR Sheffield BRC and the JP Moulton Charitable Foundation and was carried out at/supported by the NIHR Sheffield Clinical Research Facility. The views expressed are those of the author(s) and not necessarily those of the NHS, the NIHR or the Department of Health and Social Care.

TP is funded by the NIHR Sheffield BRC and The Cure Parkinson’s Trust. AA is funded by the Sheffield NIHR Sheffield BRC. The funding sources had no role in the design of this study and will not have any role during its execution, analyses, interpretation of the data, or decision to submit results.

Competing Interest

All authors declare no competing interests relating to this work.

Licence

I, the Submitting Author has the right to grant and does grant on behalf of all authors of the Work (as defined in the below author licence), an exclusive licence and/or a non-exclusive licence for contributions from authors who are: i) UK Crown employees; ii) where BMJ has agreed a CC-BY licence shall apply, and/or iii) in accordance with the terms applicable for US Federal Government officers or employees acting as part of their official duties; on a worldwide, perpetual, irrevocable, royalty-free basis to BMJ Publishing Group Ltd ("BMJ") its licensees and where the relevant Journal is co-owned by BMJ to the co-owners of the Journal, to publish the Work in BMJ Open and any other BMJ products and to exploit all rights, as set out in our licence.

The Submitting Author accepts and understands that any supply made under these terms is made by BMJ to the Submitting Author unless you are acting as an employee on behalf of your employer or a postgraduate student of an affiliated institution which is paying any applicable article publishing charge ("APC") for Open Access articles. Where the Submitting Author wishes to make the Work available on an Open Access basis (and intends to pay the relevant APC), the terms of reuse of such Open Access shall be governed by a Creative Commons licence – details of these licences and which Creative Commons licence will apply to this Work are set out in our licence referred to above.

REFERENCES

1. Kalia LV, Lang AE. Parkinson's disease. *The Lancet* 2015;386(9996):896-912. doi: 10.1016/s0140-6736(14)61393-3

2. Dorsey ER, Constantinescu R, Thompson JP, et al. Projected number of people with Parkinson disease in the most populous nations, 2005 through 2030. *Neurology* 2007;68(5):384-6. doi: 10.1212/01.wnl.0000247740.47667.03 [published Online First: 2006/11/04]

3. Schapira AHV, Olanow CW, Greenamyre JT, et al. Slowing of neurodegeneration in Parkinson's disease and Huntington's disease: future therapeutic perspectives. *The Lancet* 2014;384(9942):545-55. doi: 10.1016/s0140-6736(14)61010-2

4. Mortiboys H, Aasly J, Bandmann O. Ursocholanic acid rescues mitochondrial function in common forms of familial Parkinson's disease. *Brain* 2013;136(Pt 10):3038-50. doi: 10.1093/brain/awt224 [published Online First: 2013/09/04]

5. Mortiboys H, Furmston R, Bronstad G, et al. UDCA exerts beneficial effect on mitochondrial dysfunction in LRRK2(G2019S) carriers and in vivo. *Neurology* 2015;85(10):846-52. doi: 10.1212/WNL.0000000000001905 [published Online First: 2015/08/09]

6. Abdelkader NF, Safar MM, Salem HA. Ursodeoxycholic Acid Ameliorates Apoptotic Cascade in the Rotenone Model of Parkinson's Disease: Modulation of Mitochondrial Perturbations. *Mol Neurobiol* 2016;53(2):810-7. doi: 10.1007/s12035-014-9043-8 [published Online First: 2014/12/17]

7. Castro-Caldas M, Carvalho AN, Rodrigues E, et al. Tauroursodeoxycholic acid prevents MPTP-induced dopaminergic cell death in a mouse model of

For peer review only

Parkinson's disease. *Mol Neurobiol* 2012;46(2):475-86. doi: 10.1007/s12035-012-8295-4 [published Online First: 2012/07/10]

8. Timmons S, Coakley MF, Moloney AM, et al. Akt signal transduction dysfunction in Parkinson's disease. *Neurosci Lett* 2009;467(1):30-5. doi: 10.1016/j.neulet.2009.09.055 [published Online First: 2009/10/06]

9. Malagelada C, Jin ZH, Greene LA. RTP801 is induced in Parkinson's disease and mediates neuron death by inhibiting Akt phosphorylation/activation. *J Neurosci* 2008;28(53):14363-71. doi: 10.1523/JNEUROSCI.3928-08.2008 [published Online First: 2009/01/02]

10. Goulis J, Leandro G, Burroughs AK. Randomised controlled trials of ursodeoxycholic-acid therapy for primary biliary cirrhosis: a meta-analysis. *The Lancet* 1999;354(9184):1053-60. doi: 10.1016/s0140-6736(98)11293-x

11. Cullen SN, Rust C, Fleming K, et al. High dose ursodeoxycholic acid for the treatment of primary sclerosing cholangitis is safe and effective. *J Hepatol* 2008;48(5):792-800. doi: 10.1016/j.jhep.2007.12.023 [published Online First: 2008/03/04]

12. Ward A, Brogden RN, Heel RC, et al. Ursodeoxycholic acid: a review of its pharmacological properties and therapeutic efficacy. *Drugs* 1984;27(2):95-131. doi: 10.2165/00003495-198427020-00001 [published Online First: 1984/02/01]

13. Parry GJ, Rodrigues CM, Aranha MM, et al. Safety, tolerability, and cerebrospinal fluid penetration of ursodeoxycholic Acid in patients with amyotrophic lateral sclerosis. *Clinical neuropharmacology* 2010;33(1):17-21. doi: 10.1097/WNF.0b013e3181c47569 [published Online First: 2009/11/26]

For peer review only

14. Hughes AJ, Daniel S, Kilford L, et al. Accuracy of clinical diagnosis of idiopathic Parkinson's disease: a clinico-pathological study of 100 cases. *J Neurol Neurosurg Psychiatry* 1992;55(3):181-84.

15. Athauda D, Maclagan K, Skene SS, et al. Exenatide once weekly versus placebo in Parkinson's disease: a randomised, double-blind, placebo-controlled trial. *The Lancet* 2017;390(10103):1664-75. doi: 10.1016/s0140-6736(17)31585-4

16. Nasreddine ZS, Phillips NA, Bedirian V, et al. The Montreal Cognitive Assessment, MoCA: a brief screening tool for mild cognitive impairment. *Journal of the American Geriatrics Society* 2005;53(4):695-9. doi: 10.1111/j.1532-5415.2005.53221.x [published Online First: 2005/04/09]

17. Montgomery SA, Asberg M. A new depression scale designed to be sensitive to change. *Br J Psychiatry* 1979;134:382-9. doi: 10.1192/bjp.134.4.382 [published Online First: 1979/04/01]

18. Goetz CG, Tilley BC, Shaftman SR, et al. Movement Disorder Society-sponsored revision of the Unified Parkinson's Disease Rating Scale (MDS-UPDRS): scale presentation and clinimetric testing results. *Mov Disord* 2008;23(15):2129-70. doi: 10.1002/mds.22340 [published Online First: 2008/11/26]

19. Jenkinson C, Fitzpatrick R, Peto V, et al. The Parkinson's Disease Questionnaire (PDQ-39): development and validation of a Parkinson's disease summary index score. *Age and ageing* 1997;26(5):353-7. doi: 10.1093/ageing/26.5.353 [published Online First: 1997/11/14]

20. Chaudhuri KR, Martinez-Martin P, Schapira AH, et al. International multicenter pilot study of the first comprehensive self-completed nonmotor symptoms questionnaire for Parkinson's disease: the NMSQuest study. *Mov Disord*

For peer review only

2006;21(7):916-23. doi: 10.1002/mds.20844 [published Online First: 2006/03/21]

21. Abbas G, Lindor KD. Pharmacological treatment of biliary cirrhosis with ursodeoxycholic acid. *Expert opinion on pharmacotherapy* 2010;11(3):387-92. doi: 10.1517/14656560903493460 [published Online First: 2010/01/28]

22. Blauwendraat C, Faghri F, Pihlstrom L, et al. NeuroChip, an updated version of the NeuroX genotyping platform to rapidly screen for variants associated with neurological diseases. *Neurobiol Aging* 2017;57:247 e9-47 e13. doi: 10.1016/j.neurobiolaging.2017.05.009 [published Online First: 2017/06/13]

23. Velseboer DC, de Bie RM, Wieske L, et al. Development and external validation of a prognostic model in newly diagnosed Parkinson disease. *Neurology* 2016;86(11):986-93. doi: 10.1212/WNL.0000000000002437 [published Online First: 2016/02/19]

24. Horvath K, Aschermann Z, Acs P, et al. Minimal clinically important difference on the Motor Examination part of MDS-UPDRS. *Parkinsonism Relat Disord* 2015;21(12):1421-6. doi: 10.1016/j.parkreldis.2015.10.006 [published Online First: 2015/11/19]

25. Holden SK, Finseth T, Sillau SH, et al. Progression of MDS-UPDRS Scores Over Five Years in De Novo Parkinson Disease from the Parkinson's Progression Markers Initiative Cohort. *Mov Disord Clin Pract* 2018;5(1):47-53. doi: 10.1002/mdc3.12553 [published Online First: 2018/04/18]

26. Ketharanathan T, Hanwella R, Weerasundera R, et al. Diagnostic Validity and Factor Analysis of Montgomery-Asberg Depression Rating Scale in Parkinson Disease Population. *Journal of geriatric psychiatry and neurology*

For peer review only

2016;29(3):115-9. doi: 10.1177/0891988715606232 [published Online First: 2015/09/24]

27. Tomlinson CL, Stowe R, Patel S, et al. Systematic review of levodopa dose equivalency reporting in Parkinson's disease. *Mov Disord* 2010;25(15):2649-53. doi: 10.1002/mds.23429 [published Online First: 2010/11/12]

28. Iles RA, Stevens AN, Griffiths JR, et al. Phosphorylation status of liver by 31P-n.m.r. spectroscopy, and its implications for metabolic control. A comparison of 31P-n.m.r. spectroscopy (in vivo and in vitro) with chemical and enzymic determinations of ATP, ADP and Pi. *Biochem J* 1985;229(1):141-51. [published Online First: 1985/07/01]

29. Ordidge RJ, Connelly A, Lohman JAB. Image-selected in Vivo spectroscopy (ISIS). A new technique for spatially selective nmr spectroscopy. *Journal of Magnetic Resonance (1969)* 1986;66(2):283-94. doi: https://doi.org/10.1016/0022-2364(86)90031-4

30. Ordidge RJ, Bowley RM, McHale G. A general approach to selection of multiple cubic volume elements using the ISIS technique. *Magnetic Resonance in Medicine* 1988;8(3):323-31. doi: 10.1002/mrm.1910080309

31. Hattingen E, Magerkurth J, Pilatus U, et al. Phosphorus and proton magnetic resonance spectroscopy demonstrates mitochondrial dysfunction in early and advanced Parkinson's disease. *Brain* 2009;132(Pt 12):3285-97. doi: 10.1093/brain/awp293 [published Online First: 2009/12/03]

32. Hu MTM, Taylor-Robinson SD, Chaudhuri KR, et al. Cortical dysfunction in non-demented Parkinson's disease patients: A combined 31P-MRS and 18FDG-PET study. *Brain* 2000;123(2):340-52. doi: 10.1093/brain/123.2.340

33. Vanhamme L, van den Boogaart A, Van Huffel S. Improved Method for Accurate and Efficient Quantification of MRS Data with Use of Prior Knowledge. *Journal of Magnetic Resonance* 1997;129(1):35-43. doi: 10.1006/jmre.1997.1244

34. Stefan D, Cesare FD, Andrasescu A, et al. Quantitation of magnetic resonance spectroscopy signals: the jMRUI software package. *Measurement Science and Technology* 2009;20(10) doi: 10.1088/0957-0233/20/10/104035

35. Naressi A, Couturier C, Devos JM, et al. Java-based graphical user interface for the MRUI quantitation package. *Magnetic Resonance Materials in Physics, Biology and Medicine* 2001;12(2):141. doi: 10.1007/BF02668096

36. Podsiadlo D, Richardson S. The Timed "Up & Go": A Test of Basic Functional Mobility for Frail Elderly Persons. *Journal of the American Geriatrics Society* 1991;39(2):142-48. doi: 10.1111/j.1532-5415.1991.tb01616.x

37. van Lummel RC, Walgaard S, Hobert MA, et al. Intra-Rater, Inter-Rater and Test-Retest Reliability of an Instrumented Timed Up and Go (iTUG) Test in Patients with Parkinson's Disease. *PLoS One* 2016;11(3):e0151881. doi: 10.1371/journal.pone.0151881 [published Online First: 2016/03/22]

38. Buckley C, Galna B, Rochester L, et al. Upper body accelerations as a biomarker of gait impairment in the early stages of Parkinson's disease. *Gait & Posture* 2019;71:289-95. doi: https://doi.org/10.1016/j.gaitpost.2018.06.166

39. Rehman RZU, Del Din S, Buckley C, et al. Accelerometry-Based Digital Gait Characteristics for Classification of Parkinson's Disease: What Counts? *IEEE Open Journal of Engineering in Medicine and Biology* 2020;1:65-73. doi: 10.1109/ojemb.2020.2966295

For peer review only

40. Del Din S, Godfrey A, Mazza C, et al. Free-living monitoring of Parkinson's disease: Lessons from the field. *Mov Disord* 2016;31(9):1293-313. doi: 10.1002/mds.26718 [published Online First: 2016/07/28]

41. Buckley C, Alcock L, McArdle R, et al. The Role of Movement Analysis in Diagnosing and Monitoring Neurodegenerative Conditions: Insights from Gait and Postural Control. *Brain Sci* 2019;9(2) doi: 10.3390/brainsci9020034 [published Online First: 2019/02/10]

42. Mortiboys H, Johansen KK, Aasly JO, et al. Mitochondrial impairment in patients with Parkinson disease with the G2019S mutation in LRRK2. *Neurology* 2010;75(22):2017-20. doi: 10.1212/WNL.0b013e3181ff9685 [published Online First: 2010/12/01]

43. Di Maio R, Barrett PJ, Hoffman EK, et al. alpha-Synuclein binds to TOM20 and inhibits mitochondrial protein import in Parkinson's disease. *Sci Transl Med* 2016;8(342):342ra78. doi: 10.1126/scitranslmed.aaf3634 [published Online First: 2016/06/10]

44. Sathe AG, Tuite P, Chen C, et al. Pharmacokinetics, Safety, and Tolerability of Orally Administered Ursodeoxycholic Acid in Patients With Parkinson's Disease-A Pilot Study. *J Clin Pharmacol* 2020 doi: 10.1002/jcph.1575 [published Online First: 2020/02/14]

45. Weiss A, Sharifi S, Plotnik M, et al. Toward Automated, At-Home Assessment of Mobility Among Patients With Parkinson Disease, Using a Body-Worn Accelerometer. *Neurorehabilitation and Neural Repair* 2011;25(9):810-18. doi: 10.1177/1545968311424869

For peer review only

46. Morris R, Hickey A, Del Din S, et al. A model of free-living gait: A factor analysis in Parkinson's disease. *Gait & Posture* 2017;52:68-71. doi: https://doi.org/10.1016/j.gaitpost.2016.11.024

Key Inclusion Criteria
• Diagnosis of Parkinson's disease ≤ 3 years ago based on Queen Square Brain Bank criteria [14]<br>• Subjective improvement of motor impairment on dopaminergic medication with confirmation by a movement disorders expert<br>• Hoehn and Yahr stage ≤ 2.5 in the practically defined "ON" medication state<br>• Age 18-75 years of any gender<br>• Able to comply with study protocol and willing to attend necessary study visits<br>• Ability to communicate in English<br>• Ability to take study drug
**Key Exclusion Criteria**
• Diagnosis or suspicion of other cause of parkinsonism<br>• Known abnormality on CT or MRI brain imaging considered likely to compromise compliance with ^{31}Phosphorus MR Spectroscopy acquisition<br>• Known claustrophobia or other reasons why patient could not tolerate or be suitable for MRI<br>• Current or previous exposure to UDCA<br>• Current or previous diagnosis of liver disease (including biliary obstruction), in particular PBC judged to be significant<br>• Prior intracerebral surgical intervention for PD (including deep-brain stimulation)<br>• Already actively participating in a trial of a device, drug or surgical treatment for PD<br>• Participants who lack the capacity to give informed consent<br>• History of alcoholism<br>• Women of child-bearing potential or pregnancy<br>• Concurrent severe depression defined by a score >16 on the Montgomery-Asberg Depression Rating Scale (MADRS)<br>• Concurrent dementia defined by a score lower than 25 on the Montreal Cognitive assessment<br>• Any medical or psychiatric condition which in the investigator's opinion compromises the potential participant's ability to participate<br>• Serum transaminases more than 2 times upper limit of normal<br>• Patients on cyclosporin, nitrendipine or dapsone<br>• Participants with previous or current diagnosis of inflammatory bowel disease

Table 1: Key Inclusion and Exclusion Criteria for The UP Study

For peer review only

Figure 1: Representative ^{31}P-MRS spectra obtained from the midbrain of a healthy volunteer following appropriate phasing and 10Hz Lorentzian apodization. From left to right, phosphomonoesters (PME), inorganic phosphate (Pi), phosphodiesters (PDE), phosphocreatine (PCr), and the three spectral resonances of adenosine triphosphate (γ-,α-,β-ATP).

Figure 2: The substantia nigra slice is placed to cover the midbrain with the highlighted voxels of interest for subsequent analyses highlighted in yellow in the sagittal (A) and axial planes (B).Placement of ^{31}P-MRS slices. The basal ganglia slice is placed over the putamen aligned in both the coronal (C) axial planes (D), and voxels of interest for subsequent analyses are highlighted in yellow. One voxel covers the anterior putamen and another the posterior putamen.

Figure 3: Protocols deployed at the two sites. All participants undergo seven day physical activity monitoring in order to estimate physical activity levels and capture temporal and gait quality measures in a real-world setting. In-clinic instrumented gait tasks are also completed at both sites to provide spatiotemporal and gait quality measures of gait capacity. At UCL only red sensor location is implemented.

For peer review only

For peer review only

	Procedure	Screening	Baseline	Week 12	Week 24	Week 36	Week 48	Week 56
Medical History	Consent	X						
	Review inclusion/exclusion criteria	X	X					
	Demographics	X						
	Medical History and Physical Examination	X						
	Height and Weight	X					X	
	Genetics Sample		X					
Medication	Randomisation		X					
	Medication supply		X	X	X	X		
	Concomitant medication review	X	X	X	X	X	X	X
	Compliance review			X	X	X		
Clinical Assessment/Outcome Measures	MDS-UPDRS Part 3 'OFF'		X				X	X
	MDS-UPDRS Part 3 'ON'				X			
	MDS-UPDRS Parts 1-4 'ON'		X				X	X
	MoCA, MADRS	X					X	X
	PDQ-39		X				X	X
	NMS -QUEST		X				X	
Sensor Based Analysi	Dynaport MoveMonitor+ 7 day recording	X					X (7 days prior)	
	OptoGait/Opals gait assessment 'OFF'		X				X	
MRI	31P-MRS		X				X	
Safety Monitoring	Safety bloods	X	X	X	X	X	X	X
	ECG	X			X			
	AE Review		X	X	X	X	X	X

Table 2: Schedule of activities for The UP Study

For peer review only

Sequence description	Localisation	Decoupling, NOE	TR (ms)	TE (ms)	NSA	Acquired voxel size	Reconstruction matrix	Reconstructed voxel size	Scan duration (min)
^{31}P-Basal Ganglia	^{31}P 2D CSI ISIS localisation	On	4000	0.22	10	40x40x20	12x12	17.5x17.5x20	12:48
^{31}P-Substantia Nigra	^{31}P 2D CSI ISIS localisation	On	4000	0.22	8	40x40x20	14x14	15x15x20	10:16

Table 3: Detailed parameters of the ^{31}P protocol for acquisition. NOE; Nuclear Overhauser Effect, TR; time to repetition, TE; time to echo, NSA; number of signal averages

For peer review only

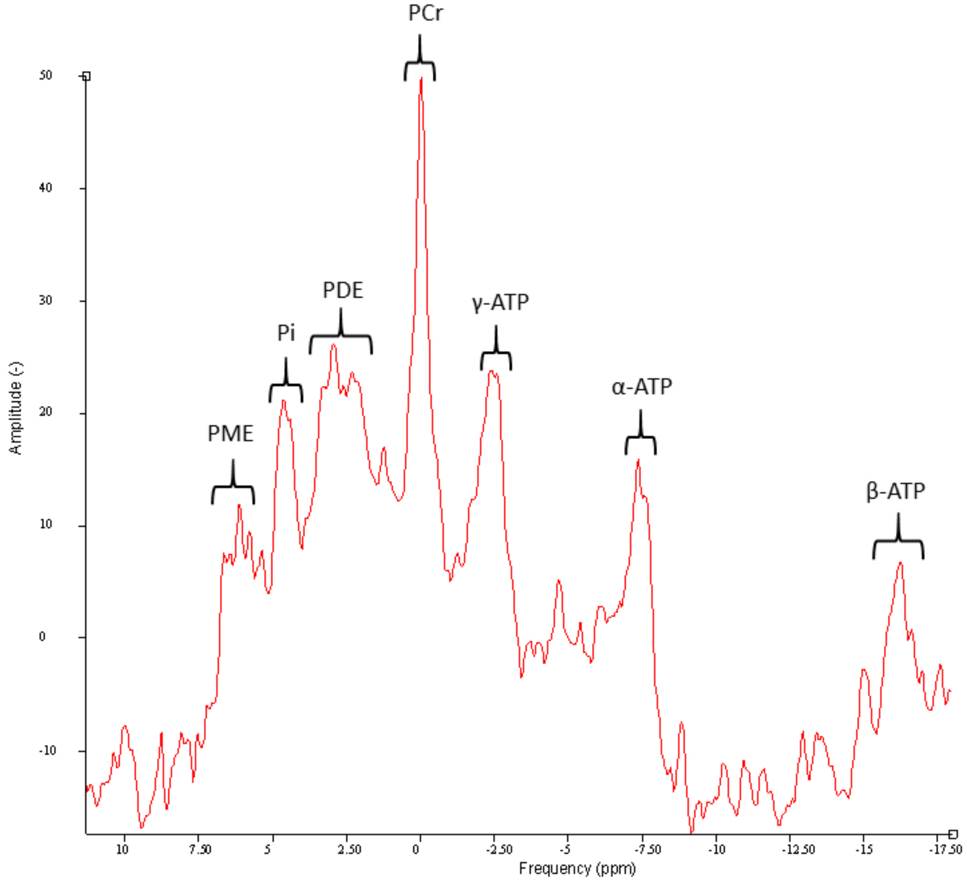

Figure 1: Representative 31P-MRS spectra obtained from the midbrain of a healthy volunteer following appropriate phasing and 10Hz Lorentzian apodization. From left to right, phosphomonoesters (PME), inorganic phosphate (Pi), phosphodiesters (PDE), phosphocreatine (PCr), and the three spectral resonances of adenosine triphosphate (γ-,α-,β-ATP).

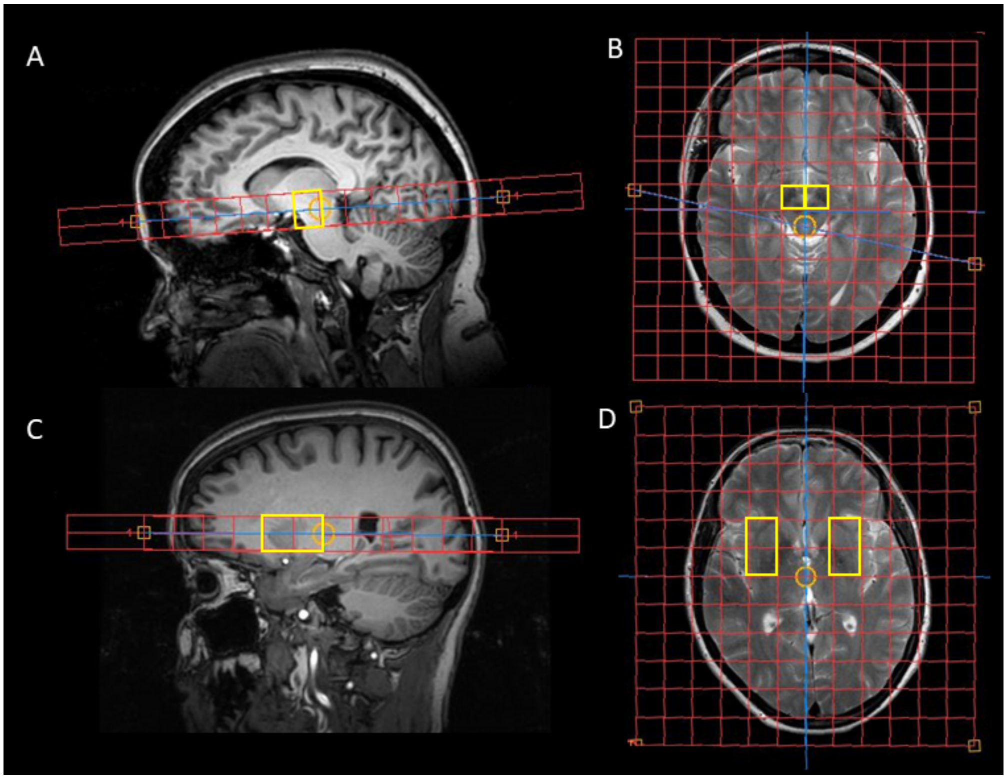

Figure 2: The substantia nigra slice is placed to cover the midbrain with the highlighted voxels of interest for subsequent analyses highlighted in yellow in the sagittal (A) and axial planes (B).Placement of 31P-MRS slices. The basal ganglia slice is placed over the putamen aligned in both the coronal (C) axial planes (D), and voxels of interest for subsequent analyses are highlighted in yellow. One voxel covers the anterior putamen and another the posterior putamen.

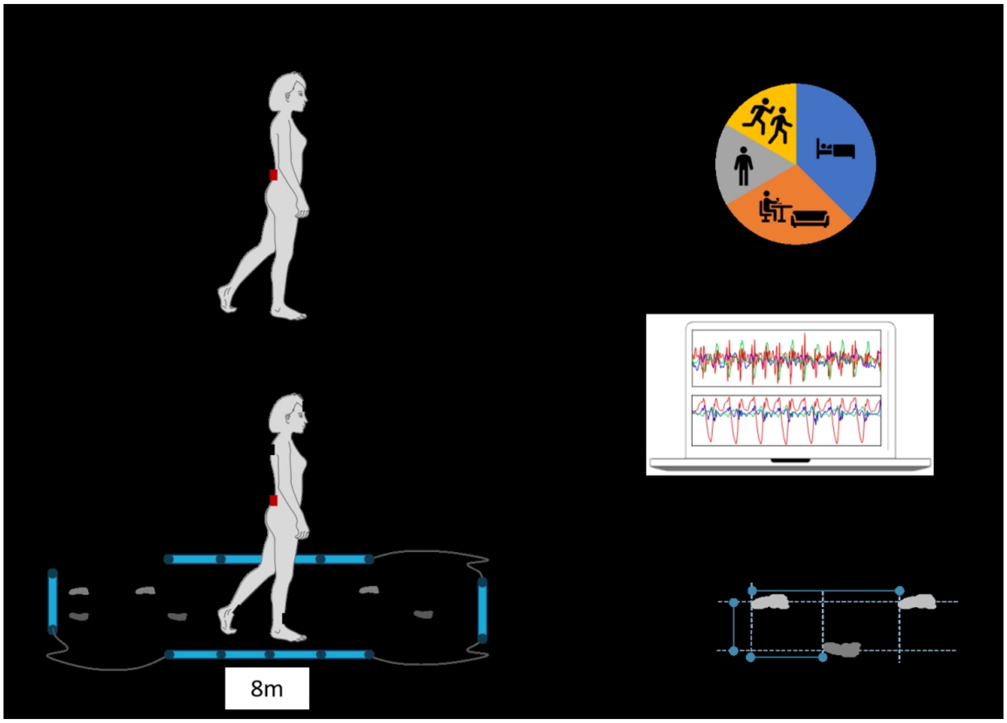

Figure 3: Protocols deployed at the two sites. All participants undergo seven day physical activity monitoring in order to estimate physical activity levels and capture temporal and gait quality measures in a real-world setting. In-clinic instrumented gait tasks are also completed at both sites to provide spatiotemporal and gait quality measures of gait capacity. At UCL only red sensor location is implemented.

249x178mm (150 x 150 DPI)

< Local Headers to be added >

Study Title: A Phase II, Placebo Controlled, Double Blind, Randomised Clinical Trial to Assess the Safety and Tolerability of 30 mg/KG Daily **U**rsodeoxycholic Acid (UDCA) In Patients With **P**arkinson's Disease (PD).

The "**UP**- Study"

Names of researchers: Prof Oliver Bandmann, Professor of Movement Disorders Neurology, Sheffield Institute for Translational Neurosciences. Prof Tom Foltynie, Consultant Neurologist & Professor of Clinical Neurology, University College London.

STH Project Number: STH18493 Patient ID number: ________________________

PATIENT CONSENT FORM

Please initial all boxes

1. I confirm that I have read and understand the information sheet dated ………… (Version …….) for the above study. ☐
2. I have had the opportunity to consider the information, ask questions and have had these answered satisfactorily. ☐
3. I understand that my participation is voluntary and that I am free to withdraw at any time without giving any reason, without my medical care or legal rights being affected. ☐
4. I understand that relevant sections of my medical notes and data collected during the study may be looked at by researchers, individuals from regulatory authorities or from the NHS Trust, where it is relevant to my taking part in this research. I give permission for these individuals to have access to my records. ☐
5. I understand and agree that my blood sample will be used for genetic analysis to help us understand whether genetic changes may influence how patients respond to treatment with UDCA. ☐
6. I agree for my anonymised samples to be used in future research (where the research project has the appropriate approvals in place). ☐
7. I agree to my GP and/ or consultant being informed of my participation in this study and if there are any significant results found as a result of taking part in this study. ☐
8. I agree to have an MRI and MR spectroscopy scan of my brain (at Sheffield Teaching Hospitals only) as described in the Information Sheet. ☐

Original for the researcher (filed in Investigator site file); copy to participant and copy to medical records.

For peer review only - http://bmjopen.bmj.com/site/about/guidelines.xhtml

9. I agree to participate in the gait analysis as described in the Information sheet. ☐

10. I agree to take part in the above study. ☐

Name of Participant	Date	Signature
Name of Person taking consent	Date	Signature

For peer review only

Original for the researcher (filed in Investigator site file); copy to participant and copy to medical records.

For peer review only - http://bmjopen.bmj.com/site/about/guidelines.xhtml

Reporting checklist for protocol of a clinical trial.

Based on the SPIRIT guidelines.

Instructions to authors

Complete this checklist by entering the page numbers from your manuscript where readers will find each of the items listed below.

Your article may not currently address all the items on the checklist. Please modify your text to include the missing information. If you are certain that an item does not apply, please write "n/a" and provide a short explanation.

Upload your completed checklist as an extra file when you submit to a journal.

In your methods section, say that you used the SPIRITreporting guidelines, and cite them as:

Chan A-W, Tetzlaff JM, Altman DG, Laupacis A, Gøtzsche PC, Krleža-Jerić K, Hróbjartsson A, Mann H, Dickersin K, Berlin J, Doré C, Parulekar W, Summerskill W, Groves T, Schulz K, Sox H, Rockhold FW, Rennie D, Moher D. SPIRIT 2013 Statement: Defining standard protocol items for clinical trials. Ann Intern Med. 2013;158(3):200-207

		Reporting Item	Page Number
**Administrative information**			
Title	#1	Descriptive title identifying the study design, population, interventions, and, if applicable, trial acronym	1
Trial registration	#2a	Trial identifier and registry name. If not yet registered, name of intended registry	3
Trial registration: data set	#2b	All items from the World Health Organization Trial Registration Data Set	36
Protocol version	#3	Date and version identifier	1
Funding	#4	Sources and types of financial, material, and other support	22
Roles and responsibilities: contributorship	#5a	Names, affiliations, and roles of protocol contributors	1, 22

For peer review only

Roles and responsibilities: sponsor contact information	#5b	Name and contact information for the trial sponsor	22
Roles and responsibilities: sponsor and funder	#5c	Role of study sponsor and funders, if any, in study design; collection, management, analysis, and interpretation of data; writing of the report; and the decision to submit the report for publication, including whether they will have ultimate authority over any of these activities	22
Roles and responsibilities: committees	#5d	Composition, roles, and responsibilities of the coordinating centre, steering committee, endpoint adjudication committee, data management team, and other individuals or groups overseeing the trial, if applicable (see Item 21a for data monitoring committee)	n/a
**Introduction**			
Background and rationale	#6a	Description of research question and justification for undertaking the trial, including summary of relevant studies (published and unpublished) examining benefits and harms for each intervention	5
Background and rationale: choice of comparators	#6b	Explanation for choice of comparators	8
Objectives	#7	Specific objectives or hypotheses	5
Trial design	#8	Description of trial design including type of trial (eg, parallel group, crossover, factorial, single group), allocation ratio, and framework (eg, superiority, equivalence, non-inferiority, exploratory)	7
**Methods: Participants, interventions, and outcomes**			
Study setting	#9	Description of study settings (eg, community clinic, academic hospital) and list of countries where data will be collected. Reference to where list of study sites can be obtained	7
Eligibility criteria	#10	Inclusion and exclusion criteria for participants. If applicable, eligibility criteria for study centres and individuals who will	7

For peer review only - http://bmjopen.bmj.com/site/about/guidelines.xhtml

		perform the interventions (eg, surgeons, psychotherapists)	
Interventions: description	#11a	Interventions for each group with sufficient detail to allow replication, including how and when they will be administered	10
Interventions: modifications	#11b	Criteria for discontinuing or modifying allocated interventions for a given trial participant (eg, drug dose change in response to harms, participant request, or improving / worsening disease)	13
Interventions: adherance	#11c	Strategies to improve adherence to intervention protocols, and any procedures for monitoring adherence (eg, drug tablet return; laboratory tests)	9, 13
Interventions: concomitant care	#11d	Relevant concomitant care and interventions that are permitted or prohibited during the trial	n/a
Outcomes	#12	Primary, secondary, and other outcomes, including the specific measurement variable (eg, systolic blood pressure), analysis metric (eg, change from baseline, final value, time to event), method of aggregation (eg, median, proportion), and time point for each outcome. Explanation of the clinical relevance of chosen efficacy and harm outcomes is strongly recommended	13-17
Participant timeline	#13	Time schedule of enrolment, interventions (including any run-ins and washouts), assessments, and visits for participants. A schematic diagram is highly recommended (see Figure)	10
Sample size	#14	Estimated number of participants needed to achieve study objectives and how it was determined, including clinical and statistical assumptions supporting any sample size calculations	11
Recruitment	#15	Strategies for achieving adequate participant enrolment to reach target sample size	7
**Methods: Assignment of interventions (for controlled trials)**			
Allocation: sequence generation	#16a	Method of generating the allocation sequence (eg, computer-generated random numbers), and list of any factors for stratification. To reduce predictability of a random sequence, details of any planned restriction (eg, blocking) should be provided in a separate document that is unavailable to those who enrol participants or assign interventions	9

For peer review only

Allocation concealment mechanism	#16b	Mechanism of implementing the allocation sequence (eg, central telephone; sequentially numbered, opaque, sealed envelopes), describing any steps to conceal the sequence until interventions are assigned	9
Allocation: implementation	#16c	Who will generate the allocation sequence, who will enrol participants, and who will assign participants to interventions	9
Blinding (masking)	#17a	Who will be blinded after assignment to interventions (eg, trial participants, care providers, outcome assessors, data analysts), and how	10
Blinding (masking): emergency unblinding	#17b	If blinded, circumstances under which unblinding is permissible, and procedure for revealing a participant's allocated intervention during the trial	13
**Methods: Data collection, management, and analysis**			
Data collection plan	#18a	Plans for assessment and collection of outcome, baseline, and other trial data, including any related processes to promote data quality (eg, duplicate measurements, training of assessors) and a description of study instruments (eg, questionnaires, laboratory tests) along with their reliability and validity, if known. Reference to where data collection forms can be found, if not in the protocol	13-17
Data collection plan: retention	#18b	Plans to promote participant retention and complete follow-up, including list of any outcome data to be collected for participants who discontinue or deviate from intervention protocols	17
Data management	#19	Plans for data entry, coding, security, and storage, including any related processes to promote data quality (eg, double data entry; range checks for data values). Reference to where details of data management procedures can be found, if not in the protocol	18
Statistics: outcomes	#20a	Statistical methods for analysing primary and secondary outcomes. Reference to where other details of the statistical analysis plan can be found, if not in the protocol	17
Statistics: additional analyses	#20b	Methods for any additional analyses (eg, subgroup and adjusted analyses)	17

For peer review only

Statistics: analysis population and missing data	#20c	Definition of analysis population relating to protocol non-adherence (eg, as randomised analysis), and any statistical methods to handle missing data (eg, multiple imputation)	17
**Methods: Monitoring**			
Data monitoring: formal committee	#21a	Composition of data monitoring committee (DMC); summary of its role and reporting structure; statement of whether it is independent from the sponsor and competing interests; and reference to where further details about its charter can be found, if not in the protocol. Alternatively, an explanation of why a DMC is not needed	13
Data monitoring: interim analysis	#21b	Description of any interim analyses and stopping guidelines, including who will have access to these interim results and make the final decision to terminate the trial	n/a
Harms	#22	Plans for collecting, assessing, reporting, and managing solicited and spontaneously reported adverse events and other unintended effects of trial interventions or trial conduct	13
Auditing	#23	Frequency and procedures for auditing trial conduct, if any, and whether the process will be independent from investigators and the sponsor	13
**Ethics and dissemination**			
Research ethics approval	#24	Plans for seeking research ethics committee / institutional review board (REC / IRB) approval	18
Protocol amendments	#25	Plans for communicating important protocol modifications (eg, changes to eligibility criteria, outcomes, analyses) to relevant parties (eg, investigators, REC / IRBs, trial participants, trial registries, journals, regulators)	18
Consent or assent	#26a	Who will obtain informed consent or assent from potential trial participants or authorised surrogates, and how (see Item 32)	8-9
Consent or assent: ancillary studies	#26b	Additional consent provisions for collection and use of participant data and biological specimens in ancillary studies, if applicable	n/a
Confidentiality	#27	How personal information about potential and enrolled participants will be collected, shared, and maintained in order to protect confidentiality before, during, and after the trial	18

For peer review only

Declaration of interests	#28	Financial and other competing interests for principal investigators for the overall trial and each study site	22
Data access	#29	Statement of who will have access to the final trial dataset, and disclosure of contractual agreements that limit such access for investigators	n/a
Ancillary and post trial care	#30	Provisions, if any, for ancillary and post-trial care, and for compensation to those who suffer harm from trial participation	n/a
Dissemination policy: trial results	#31a	Plans for investigators and sponsor to communicate trial results to participants, healthcare professionals, the public, and other relevant groups (eg, via publication, reporting in results databases, or other data sharing arrangements), including any publication restrictions	18
Dissemination policy: authorship	#31b	Authorship eligibility guidelines and any intended use of professional writers	22
Dissemination policy: reproducible research	#31c	Plans, if any, for granting public access to the full protocol, participant-level dataset, and statistical code	n/a
**Appendices**			
Informed consent materials	#32	Model consent form and other related documentation given to participants and authorised surrogates	n/a
Biological specimens	#33	Plans for collection, laboratory evaluation, and storage of biological specimens for genetic or molecular analysis in the current trial and for future use in ancillary studies, if applicable	n/a

The SPIRIT checklist is distributed under the terms of the Creative Commons Attribution License CC-BY-ND 3.0. This checklist was completed on 24. March 2020 using https://www.goodreports.org/, a tool made by the EQUATOR Network in collaboration with Penelope.ai

For peer review only
